# The methodological quality assessment of systematic reviews/meta-analyses of chronic prostatitis/chronic pelvic pain syndrome using AMSTAR2

**DOI:** 10.1186/s12874-023-02095-0

**Published:** 2023-11-27

**Authors:** Xin Guan, Yongfeng Lao, Jian Wang, Yanan Wang, Yanan Bai, Xiaolong Li, Shuai Liu, Zewen Li, Fuhan Li, Zhilong Dong

**Affiliations:** 1 Second Clinical Medical College, Lanzhou University, Lanzhou, China; 2 Department of Urology, Lanzhou University Second Hospital, Lanzhou, China; 3 Laboratory Medicine Center, Lanzhou University Second Hospital, Lanzhou, China

**Keywords:** Systematic review, Meta-analysis, Chronic prostatitis/chronic pelvic pain syndrome, AMSTAR2, Methodological quality, Cross-sectional study

## Abstract

**Background:**

This study aimed to assess the methodological quality of the systematic reviews/meta-analyses (SRs/MAs) of chronic prostatitis/chronic pelvic pain syndrome (CP/CPPS) using A Measurement Tool to Assess systematic Reviews (AMSTAR2) and to explore the potential influencing factors.

**Methods:**

PubMed, EMBASE and Cochrane Library databases were searched for relevant studies. AMSTAR2 was used for evaluating the methodological quality of eligible SRs/MAs. Differences between methodological characteristics of SRs/MAs were compared using chi-square tests. The intra-class correlation coefficient (ICC) was used to assess reviewer agreement in the pre-experiment. Multivariate regression analysis was used to identify potential factors affecting methodological quality.

**Results:**

A total of 45 SRs/MAs were included. After AMSTAR2 evaluation, only two (4.4%) of 45 SRs/MAs were moderate, three (6.7%) were rated as low quality, and the remainder 40 (88.9%) were rated as critically low quality. Among the 16 items of AMSTAR2, item 3 and item 10 had the poorest adherence. Item 4 received the most significant number of "Partial Yes" responses. Univariable analysis indicated that there were significant differences in methodological quality in SRs between different continents (*P* = 0.027) as well as between preregistered SRs and those that were not (*P* = 0.004). However, in multivariate analysis, there was no significant association between methodological quality and the following research characteristics: publication year, continent, whether reporting followed Preferred Reporting Items for Systematic Reviews (PRISMA), preregistration, funding support, randomized controlled trials (RCT) enrollment, whether SR was published in the Cochrane Database of Systematic Reviews (CDSR), and whether with meta-analysis. Additionally, subgroup analysis based on interventional SRs/MAs showed that continent was independently associated with the methodological quality of SRs/MAs of CP/CPPS via univariable and multivariate analysis.

**Conclusions:**

Our study demonstrates that the methodological quality of SRs/MAs of CP/CPPS was generally poor. SRs/MAs of CP/CPPS should adopt the AMSTAR2 to enhance their methodological quality.

**Supplementary Information:**

The online version contains supplementary material available at 10.1186/s12874-023-02095-0.

## Background

Chronic prostatitis/chronic pelvic pain syndrome (CP/CPPS) is the most common genitourinary disorder in men under 50 years of age [1], and it has been reported that 35–50% of men in all age groups will be affected by symptoms suggesting prostatitis during their lifetime [2]. In addition, the prevalence of CP/CPPS is estimated to range from 8.4% to 25% on different continents [3]. Based on the classification system of prostatitis syndromes established by the National Institute of Health (NIH) in 1999, CP/CPPS can be divided into two subtypes: IIIA (inflammatory), and IIIB (Noninflammatory), accounting for about 90–95% of all prostatitis cases [4]. The manifestations of CP/CPPS were heterogeneous, mainly including urogenital pain, lower urinary tract symptoms (LUTS), psychological problems, and sexual dysfunction [5, 6]. The disorder not only impairs the quality of life (QOL) of patients but often leads to severe psychosocial and economic burdens [3, 6].

Although many clinical trials, as well as systematic reviews/meta-analyses (SRs/MAs) of CP/CPPS, have been published and various treatment options have been recommended, there is still no ideal and standardized management for CP/CPPS up to now [7–10]. Therefore, CP/CPPS is still a massive challenge in clinical practice. The reason for this problem is that, on the one hand, the etiology and pathophysiology of CP/CPPS remain unclear; on the other hand, it may be due to the poor quality of clinical evidence of CP/CPPS, which leads to their limited reliability and usefulness in guiding clinical practice. Primary studies, especially randomized controlled trials (RCT), often provide the most direct and powerful evidence in medical practice, making them the "gold standard" for assessing the effectiveness and safety of medical interventions [11]. A recent study suggested that the quality of RCT reports of CP/CPPS needs to be further improved and that the results of RCT of CP/CPPS should be treated with caution [12]. Additionally, the SRs/MAs are considered vital evidence for the development of clinical practice guidelines and can inform healthcare policy as well as clinical decision-making [13, 14]. As with all original research, SRs should be also assessed for their methodological rigor, as only high-quality SRs/MAs that follow specific guidelines and report normatively can provide convincing results and conclusions [15, 16]. Researchers have developed several assessment tools to assess the methodological quality of SRs/MAs, among which A Measurement Tool to Assess Systematic Reviews (AMSTAR2) is one of the most studied and widely used tools [17]. AMSTAR was developed and validated in 2007, but it could only be used to evaluate RCT-based SRs/MAs initially [18]. As a new version, AMSTAR2 enables a more detailed and reproducible assessment of the quality of SRs/MAs of randomized controlled trials (RCT) and non-RCTs [17]. Compared with other quality assessment tools, such as the risk of bias in systematic reviews (ROBIS) [19], AMSTAR2 takes into account more information related to methodological quality [17]. Therefore, AMSTAR2 has become a standard tool for evaluating the methodological quality of SRs/MAs.

To our knowledge, no studies evaluating the methodological quality of SRs/MAs of CP/CPPS have been reported. Therefore, the primary aim of this study was to assess the methodological quality of SRs/MAs of CP/CPPS using AMSTAR2 and explore potential risk factors associated with the methodological quality of SRs/MAs of CP/CPPS. This might provide an important reference for follow-up studies and optimize the production and dissemination of clinical evidence for CP/CPPS.

## Methods

### Protocol and registration

This study has been prospectively registered on the PROSPERO platform [20, 21], and the registration number is CRD42022343957 while the full registration document can be accessed from the official website [22].

### Search strategy

We comprehensively searched the literature in PubMed, EMBASE, and the Cochrane Library database for SRs/MAs of CP/CPPS from their inception to December 31, 2022. The following keywords were used as search terms: (Prostatitis OR Prostatitides OR Chronic Prostatitis with Chronic Pelvic Pain Syndrome OR Chronic Prostatitis/Chronic Pelvic Pain Syndrome OR Chronic Pelvic Pain Syndrome OR Chronic Prostatitis OR Chronic Prostatitides OR CP/CPPS OR CPPS) AND (Meta-Analysis OR Meta Analyses OR Network Meta-Analysis OR Systematic Review OR System Review OR Evidence based review OR Evidence-based review OR System evaluation OR Systematic evaluation). The full search strategy was available in the supplementary file (Additional file 1).

### Eligibility criteria

The inclusion criteria were as follows: (1) populations: the participants who met criteria for CP/CPPS categories IIIA or IIIB according to the National Institutes Health classification [4], regardless of complications, no restrictions on age, race, the source of cases, or onset time; (2) study designs: systematic reviews with or without meta-analysis; (3) language: the studies published in Chinese or English; (4) interventions: all treatments or interventions for CP/CPPS that were included in the systematic reviews; (5) comparators: all controls involved in the systematic reviews, such as placebo control, etc.; (6) publication language: Chinese or English. The exclusion criteria were as follows: (1) conference abstracts, editorials and expert opinions, letters, conference proceedings, and case reports; (2) the subjects included in the study were not human; (3) the full text could be found and unable to provide the required data. There was a certain situation that one SR published in the Cochrane Library had also been published in another journal, we only included the SR published in the Cochrane Library because it was more comprehensive [23].

### Data selection

Endnote X9, the literature management software, was used to manage the literature search records. Two trained researchers (YNB, SL) independently reviewed all the titles and abstracts of sources for preliminary inclusion against the preset eligible criteria. Then the full text of the potentially eligible articles left at the above stage was checked for final inclusion by two trained researchers (YNB, SL) independently, and the reasons for article exclusion were recorded. To ensure the quality of literature screening and reduce the risk of bias, the screening results of each author should be blind compared with other authors. A third researcher (ZLD) resolved any disagreements arising during the pairing process through negotiation and arbitration.

### Data extraction

We designed a standardized form to extract all available data, and the two trained researchers (JW, YNW) independently extracted the data. The following data were extracted from each eligible literature: (1) general characteristics: first author, publication year, country of correspondence author, number and type of included studies, sample size, whether with meta-analysis, funding support, presence or absence of preregistration (detailed platform extracted), and reporting criteria referenced by the study (such as Preferred Reporting Items for Systematic Reviews (PRISMA), Joanna Briggs Institute (JBI) Critical Appraisal Tool, and Cochrane Handbook for systematic reviews [24–26]), etc.; (2) participants' details: category of CP/CPPS, diagnostic criterion, etc.; (3) intervention/control: intervention/control measures, drug doses, duration, routes of administration, etc.; (4) outcome indicators: scores of scales such as National Institutes of Health chronic prostatitis symptom index (NIH-CPSI) scores, International prostate symptom score (IPSS), etc., the clinical effective rate of CP/CPPS, International Index of Erectile Function (IELT), etc. A third researcher (ZLD) resolved any disagreements arising during the pairing process through negotiation and arbitration.

### Methodological quality assessment

Two trained researchers (YFL, XG) independently assessed the methodological quality of all eligible literature via the AMSTAR2 tool. AMSTAR2 tool consists of 16 items, and researchers need to evaluate each item with Yes, Partial Yes (PY), or No. When the evaluation criteria of the item are fully met, the item should be rated as a “Yes”. PY indicates that the systematic review only partly complied with the standard for the given item. If no relevant information is reported to rate an item in the system reviews, the evaluation is “No”. Furthermore, seven (items 2, 4, 7, 9, 11, 13, and 15) of 16 items are considered critical domains, corresponding to the comprehensiveness of the literature search, preparation for the review, eligibility criteria, Risk of Bias (RoB) analysis and interpretation, appropriateness of meta-analysis, and potential impact of publication bias [15, 17]. Based on weaknesses identified in critical and non-critical items, AMSTAR2 classifies the overall confidence of the results of included systematic reviews into four levels: high, moderate, low, and critically low. The supplementary file (Additional files 2 and 3) showed details of the items in the AMSTAR2 tool and the definition of the four quality classifications. A third reviewer (ZLD) settled any disagreements between reviewers through consultation and arbitration.

### Consistency evaluation

The researchers who assessed the methodological quality of included studies based on the AMSTAR2 tool have undergone systematic training at the Evidence-Based Medicine Center of Lanzhou University. The articles included in this study were evaluated afterward when the two researchers reached a good agreement (at least 90%) in the pre-experiment. We used the intraclass correlation coefficient (ICC) to assess the consistency of quantitative measurements in the pre-experiment. The ICC value for the overall score was 0.920.

### Data analysis

Stata 14.0 was used for statistical analysis. The continuous variables were described with the mean ± standard deviation. The categorical variables were described by frequencies and percentages. In this study, we explored the impact of the following factors on the quality of the included SRs/MAs of CP/CPPS: (1) publication year (year in which the included SRs/MAs of CP/CPPS were published); (2) continent (classified based on the country where the first corresponding author is located); (3) following PRISMA; (4) preregistration (preregistration on any platform such as PROSPERO and Cochrane library, etc. was considered as Yes, otherwise as No); (5) funding support; (6) whether Cochrane Database of Systematic Reviews (non-CDSR, or CDSR); (7) RCT enrollment (non-RCTs, only RCTs, or RCTs and non-RCTs); (8) whether meta-analysis was performed (without a meta-analysis, or with a meta-analysis), as previous studies have suggested that these factors may be potential factors affecting the methodological quality of SRs/Mas [15, 23, 27–32]. Chi-square tests or Fisher’s exact tests were used to compare SRs/MAs characteristics based on AMSTAR2 appraisal outcomes. Multiple regression was employed to assess potential factors that may have an impact on the methodological quality of SRs/MAS of CP/CPPS, and variance inflation factors (VIFs) were utilized to test for multicollinearity among explanatory factors. Subgroup analysis was conducted based on the type of SRs (interventional and non-interventional). *P*-value ≤ 0.05 was considered significant for all statistical tests.

## Results

### Literature search

**Fig. 1:**
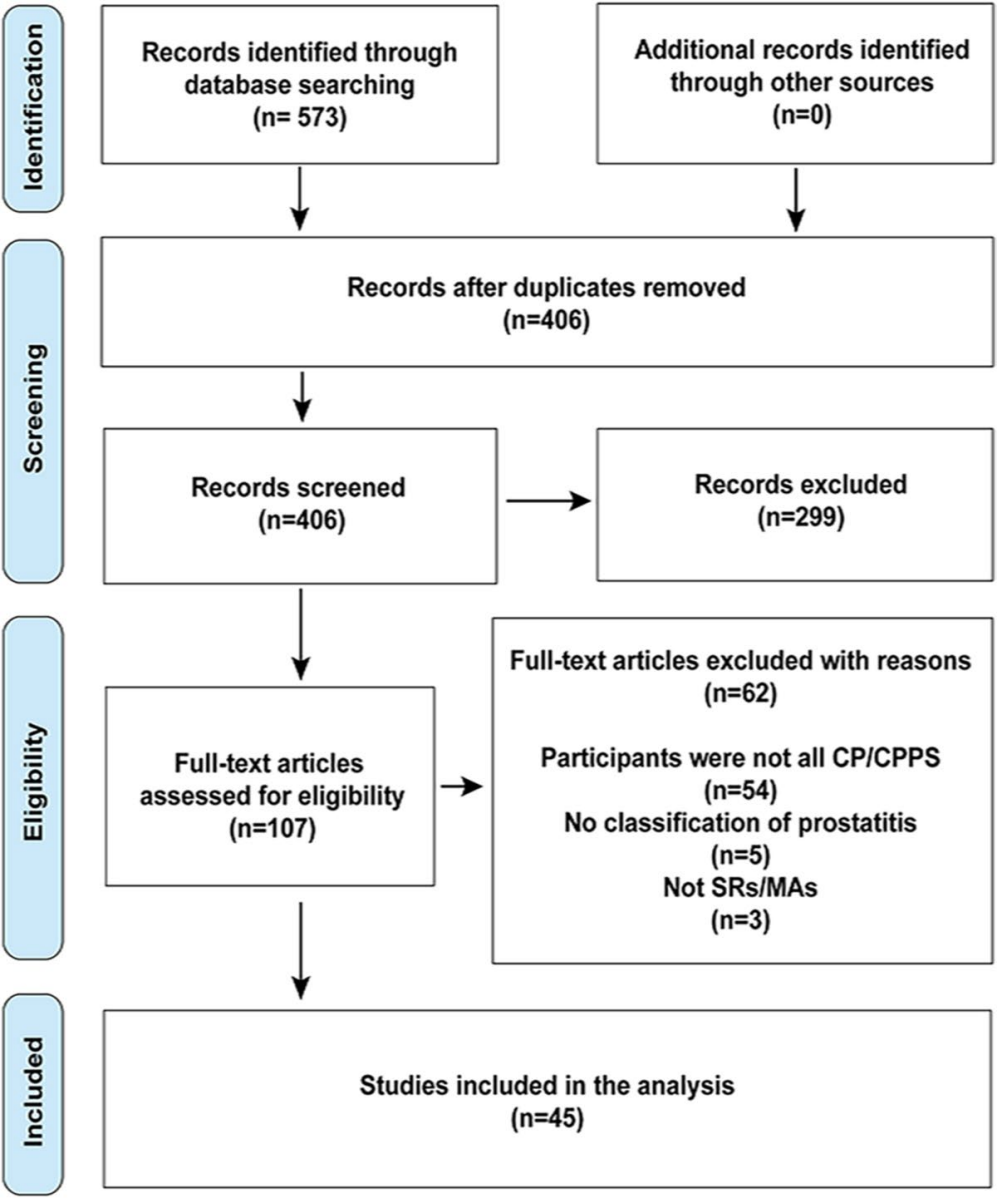
The details of the literature selection process

The process of the literature search and selection was presented in Fig. 1. A total of 573 articles were obtained after retrieval while 406 articles were left after removing duplicates. Then 299 articles were excluded after reviewing the titles and abstracts, leaving 107 records for full-text screening. Finally, 62 records were further excluded and 45 SRs/MAs were included in our study [6, 7, 33–75]. A list of the articles excluded after the screen of the full text was provided in supplement files (Additional file 4).

### Characteristics of systematic reviews

**Fig. 2:**
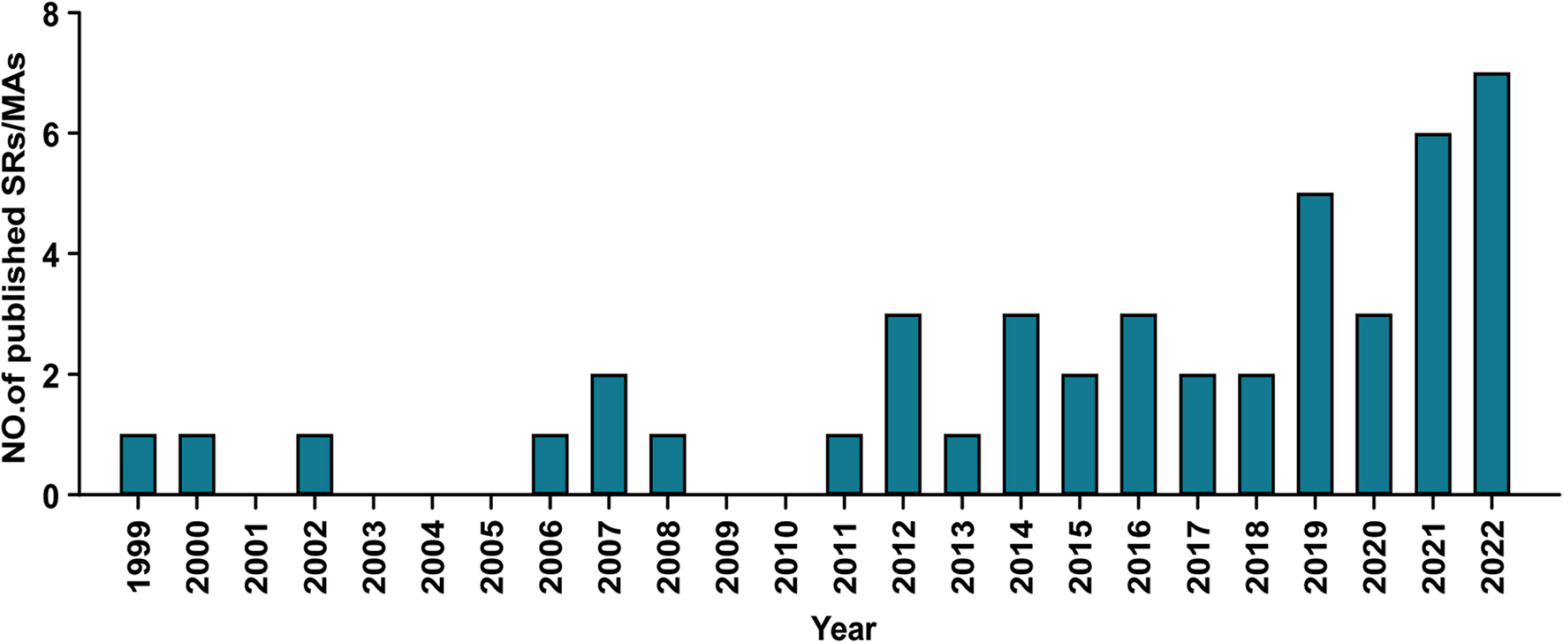
Publication year of included studies

The detailed characteristics of all included SRs/MAs are listed in Table 1. 45 SRs/MAs were published between 1999 and 2022, with no SRs/MAs of CP/CPPS published in 2001, 2003–2005, or 2009–2010, but relevant literature was published almost every year after 2010 (Fig. 2). Thus, 2010 was regarded as a time node and used for subsequent univariable and multivariable analysis since there was a burst of relative literature in the field after 2010. 44 studies enrolled patients diagnosed with CP/CPPS, while one enrolled patient diagnosed with CP/CPPS also had sexual dysfunction (SD). The minimum and maximum number of trials in the included SRs/MAs were 1 and 99 respectively, and the minimum and maximum number of participants in included studies were 54 and 44,650 respectively. The included SRs/MAs are mainly from five continents: Asia (*n* = 29, 64.4%), Europe (*n* = 6, 13.4%), North America (*n* = 8, 17.8%), and South America(*n* = 2, 4.4%). More than half of the SRs/MAs originated from China, followed by the United States (USA). Forty (88.9%) SRs/MAs were non-Cochrane reviews, and five (11.1%) SRs/MAs were Cochrane reviews. Nearly two-thirds (*n* = 29, 64.4%) of the SRs/MAs enrolled only RCTs, five (11.1%) SRs/MAs only included non-RCTs and approximately a quarter (*n* = 11, 24.4%) of SRs/MAs enrolled not only RCTs but also non-RCTs. Of all the included studies, only PRISMA was mentioned as a reporting standard for article publication. Specifically, two-thirds (*n* = 30, 66.7%) of the SRs/MAs followed the PRISMA, while the remaining did not indicate which reporting standard was referenced. Also, considering the authority and widespread use of PRISMA in SRs, we included whether PRISMA was followed or not as one of the independent variables in the subsequent risk factor analysis. Over half (*n* = 24, 53.3%) of the SRs/MAs received funding support. About one-third (*n* = 16, 35.6%) of the SRs/MAs were preregistered on platforms. Thirty-six included studies (80.0%) were systematic reviews with a meta-analysis. Thirty-eight studies (84.4%) were interventional SRs/MAs. In addition, the intervention and control measures of the included studies were shown in Additional file 5.

**Table 1:** Characteristics of included articles

Study	Country (Region)	SRs/MAs	Whether CDSR	Type of SRs	Type of included studies	Preregistration	PRISMA	Funding	Disease	Sample size
Collins et al. 1999 [33]	USA (North America)	SRs	CDSR	Interventional	RCT/Crossover trail/CCT	Y	N	Y	CP/CPPS (III)	600
Collins et al. 2000 [34]	USA (North America)	SRs	Non-CDSR	Interventional	RCT/Crossover trail/CCT	N	N	Y	CP/CPPS	1954
Collins et al. 2002 [35]	USA (North America)	SRs	CDSR	Interventional	RCT	Y	N	Y	CP/CPPS (IIIA/IIIB)	54
Yang et al. 2006 [36]	China (Asia)	MAs	Non-CDSR	Interventional	RCT	N	N	N	CP/CPPS (III)	734
Lee et al. 2007 [37]	USA (North America)	SRs	Non-CDSR	Interventional	open-label or small prospective studies/ double-blinded and placebo-controlled clinical trials	N	N	N	CP/CPPS	818
Mishra et al. 2007 [38]	UK (Europe)	SRs	Non-CDSR	Interventional	RCT	N	N	N	CP/CPPS (III)	386
Yang et al. 2008 [39]	China (Asia)	SRs/MAs	Non-CDSR	Interventional	RCT	N	N	N	CP/CPPS	1050
Anothaisintawee et al. 2011 [40]	Thailand (Asia)	SRs/MAs	Non-CDSR	Interventional	RCT	N	N	Y	CP/CPPS (IIIA/IIIB)	2021
Aboumarzouk et al. 2012 [41]	UK (Europe)	SRs	CDSR	Interventional	RCT	Y	N	Y	CP/CPPS (IIIA/IIIB)	324
Cohen et al. 2012 [42]	USA (North America)	SRs/MAs	Non-CDSR	Interventional	RCT	N	Y	N	CP/CPPS (III)	3312
Thakkinstian et al. 2012 [43]	Canada (North America)	SRs/MAs	Non-CDSR	Interventional	RCT	N	N	N	CP/CPPS (IIIA/IIIB)	1669
Moldwin et al. 2013 [44]	USA (North America)	SRs	Non-CDSR	Interventional	RCT/NRSI	N	N	N	CP/CPPS	NR
Fu et al. 2014 [45]	China (Asia)	SRs/MAs	Non-CDSR	Non-interventional	case–control studies	N	Y	N	CP/CPPS	1454
Riegel et al. 2014 [46]	Germany (Europe)	SRs	Non-CDSR	Non-interventional	RCT/prospective study/Retrospective review/Case–control study/Experimental study/Evaluation of clinical data/Longitudinal study/cohort study	Y	Y	Y	CP/CPPS (IIIA/IIIB)	44,650
Zhu et al. 2014 [47]	China (Asia)	MAs	Non-CDSR	Interventional	RCT	N	N	N	CP/CPPS (III)	539
Chen et al. 2015 [48]	China (Asia)	SRs/MAs	Non-CDSR	Non-interventional	Case–control studies/cohort studies/cross-sectional studies	Y	Y	N	CP/CPPS	33,033
Li et al. 2015 [49]	China (Asia)	MAs	Non-CDSR	Non-interventional	Observational studies/cohort studies/cross-sectional studies/case series	N	N	N	CP/CPPS	11,189
Liu et al. 2016 [50]	China (Asia)	SRs/MAs	Non-CDSR	Interventional	RCT	N	Y	N	CP/CPPS	754
Qin et al. 2016a [51]	China (Asia)	MAs	Non-CDSR	Interventional	RCT	N	Y	N	CP/CPPS (III)	1203
Qin et al. 2016b [52]	China (Asia)	SRs/MAs	Non-CDSR	Interventional	RCT	Y	Y	N	CP/CPPS (III)	471
Cai et al. 2017 [53]	Italy (Europe)	SRs/MAs	Non-CDSR	Interventional	pre-clinical studies/clinical trials/RCT/cohort studies/case–control studies	N	Y	N	CP/CPPS	590
Chang et al. 2017 [54]	China (Asia)	SRs/MAs	Non-CDSR	Interventional	RCT	N	Y	Y	CP/CPPS (IIIA/IIIB)	502
Anderson et al. 2018 [55]	USA (North America)	MAs	Non-CDSR	Interventional	RCT/case series	N	Y	N	CP/CPPS	380
Franco et al. 2018 [56]	Argentina (South America)	SRs/MAs	CDSR	Interventional	RCT	Y	Y	N	CP/CPPS (III)	3290
Franco et al. 2019 [57]	Argentina (South America)	SRs/MAs	CDSR	Interventional	RCT	Y	Y	N	CP/CPPS (III)	9119
Liao et al. 2019 [58]	China (Asia)	MAs	Non-CDSR	Interventional	RCT	N	N	Y	CP/CPPS	838
Qin et al. 2019a [59]	China (Asia)	MAs	Non-CDSR	Interventional	RCT/case series	N	Y	Y	CP/CPPS	329
Qin et al. 2019b [60]	China (Asia)	SRs/MAs	Non-CDSR	Interventional	RCT/case series	N	Y	Y	CP/CPPS	439
Yuan et al. 2019 [61]	China (Asia)	SRs/MAs	Non-CDSR	Interventional	RCT	N	N	Y	CP/CPPS	280
Birowo et al. 2020 [62]	Indonesia (Asia)	SRs/MAs	Non-CDSR	Interventional	RCT	Y	Y	Y	CP/CPPS	137
Huang et al. 2020 [63]	China (Asia)	MAs	Non-CDSR	Non-interventional	cross-sectional studies	N	Y	Y	CP/CPPS	1308
Li et al. 2020 [64]	China (Asia)	SRs/MAs	Non-CDSR	Interventional	RCT	N	Y	Y	CP/CPPS (III)	748
Chen et al. 2021 [65]	China (Asia)	SRs/MAs	Non-CDSR	Non-interventional	RCT/NRSI	N	Y	Y	CP/CPPS	NR
Kang et al. 2021 [66]	China (Asia)	MAs	Non-CDSR	Interventional	RCT	N	Y	Y	CP/CPPS	525
Li et al. 2021 [67]	China (Asia)	SRs/MAs	Non-CDSR	Interventional	RCT/Non-RCT	N	Y	Y	CPPS	317
Mykoniatis et al. 2021 [68]	Greece (Europe)	SRs/MAs	Non-CDSR	Interventional	RCT	Y	Y	N	CP/CPPS (IIIB)	316
Zhang et al. 2021a [69]	China (Asia)	MAs	Non-CDSR	Interventional	RCT	N	Y	Y	CP/CPPS (IIIA/IIIB)	770
Zhang et al. 2021b [70]	China (Asia)	MAs	Non-CDSR	Interventional	RCT	Y	Y	Y	CP/CPPS with SD	2996
Kong et al. 2022 [71]	China (Asia)	SRs/MAs	Non-CDSR	Interventional	RCT	Y	Y	Y	CP/CPPS	651
Lao et al. 2022 [72]	China (Asia)	SRs/MAs	Non-CDSR	Interventional	RCT	Y	Y	Y	CP/CPPS (IIIA/IIIB)	4244
Lok et al. 2022 [73]	China (Asia)	SRs/MAs	Non-CDSR	Interventional	RCT	N	Y	N	CP/CPPS	434
Andrey et al. 2022 [6]	Russia (Europe)	SRs/MAs	Non-CDSR	Interventional	RCT	Y	Y	N	CP/CPPS	5512
Qin et al. 2022a [7]	China (Asia)	SRs/MAs	Non-CDSR	Interventional	RCT	Y	Y	Y	CP/CPPS	1188
Qin et al. 2022b [74]	China (Asia)	SRs/MAs	Non-CDSR	Interventional	RCT	Y	Y	Y	CP/CPPS	3514
Zhao et al. 2022 [75]	China (Asia)	SRs	Non-CDSR	Non-interventional	RCT/Non-RCT	N	Y	Y	CP/CPPS (III)	431

*Y* yes, *N* no, *NR* Not reported, *CDSR*, Cochrane Database of Systematic Reviews

### Methodological quality

**Fig. 3:**
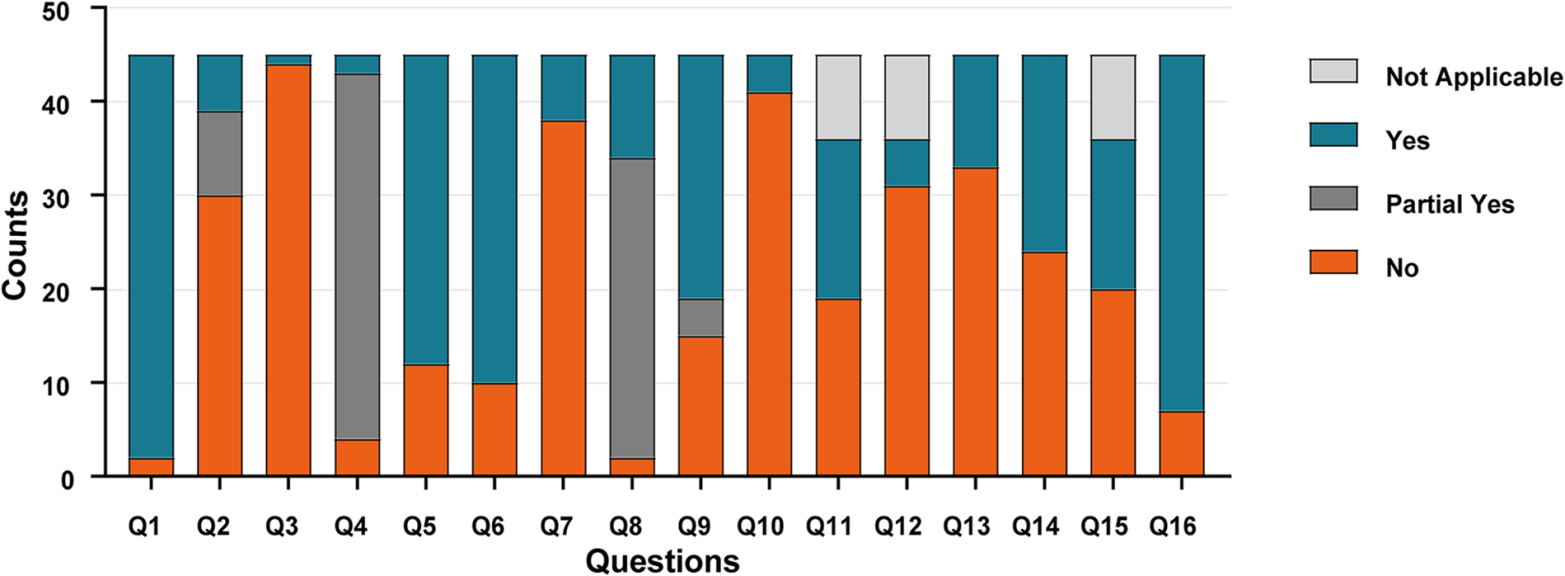
Probability of AMSTAR2 response per question

The evaluation results of all AMSTAR2 items assessment for each study are presented in Table 2 while the result distribution of each item of AMSTAR2 was shown in Fig. 3. None of the included studies was classified as high quality, two (4.4%) SRs/MAs were rated as moderate quality, three (6.7%) were assessed as low-quality, and remaining forty (88.9%) were assessed as low quality. The AMSTAR2 adherence varies widely among the items. Item 1 (PICO: populations, interventions, comparisons, and outcomes) has the best adherence in all included SRs/MAs, followed by item 16 (conflict of interest). Item 3 (selection of the study designs) and item 10 (funding reported for individual studies) showed the poorest adherence in the SRs/MAs. The most PY ratings were given on item 4 (comprehensiveness of literature strategy). Among the seven essential items, the most frequently lacking items were as follows: item 7 (*n* = 38, 84.4%), lack of excluded trials list and reasons for exclusion), item 13 (*n* = 33, 73.3%), lack of adequate discussion of the impact of risk of bias on study results), item 2 (*n* = 30, 66.7%), lack of registration before the commencement of the review).

**Table 2:** Result of the AMSTAR2 assessments

Study	AMSTAR-2																Quality
	Q1	Q2	Q3	Q4	Q5	Q6	Q7	Q8	Q9	Q10	Q11	Q12	Q13	Q14	Q15	Q16	
Collins et al. 1999 [33]	Y	Y	N	PY	Y	Y	N	Y	N	N	NA	NA	N	Y	NA	Y	CL
Collins et al. 2000 [34]	Y	N	N	PY	N	Y	N	PY	N	N	NA	NA	N	Y	NA	N	CL
Collins et al. 2002 [35]	Y	N	N	PY	Y	Y	Y	Y	N	N	NA	NA	N	Y	NA	Y	CL
Yang et al. 2006 [36]	Y	N	N	PY	N	N	N	PY	PY	N	Y	N	N	N	N	N	CL
Lee et al. 2007 [37]	Y	N	N	N	N	N	N	PY	N	N	NA	NA	N	N	NA	N	CL
Mishra et al. 2007 [38]	Y	N	N	PY	N	N	Y	Y	N	N	NA	NA	Y	Y	NA	N	CL
Yang et al. 2008 [39]	Y	N	N	PY	Y	Y	N	PY	PY	N	N	N	N	N	N	N	CL
Anothaisintawee et al. 2011 [40]	Y	N	N	PY	Y	Y	N	PY	Y	N	Y	Y	Y	Y	Y	Y	CL
Aboumarzouk et al. 2012 [41]	Y	Y	N	PY	Y	Y	Y	PY	Y	N	NA	NA	Y	Y	NA	Y	M
Cohen et al. 2012 [42]	Y	N	N	PY	Y	Y	N	Y	Y	Y	Y	N	N	Y	Y	Y	CL
Thakkinstian et al. 2012 [43]	Y	N	N	PY	N	Y	N	PY	N	N	N	N	N	Y	N	Y	CL
Moldwin et al. 2013 [44]	N	N	N	N	N	N	N	N	N	N	NA	NA	N	N	NA	Y	CL
Fu et al. 2014 [45]	Y	N	N	PY	Y	Y	N	Y	Y	N	Y	N	N	Y	N	Y	CL
Riegel et al. 2014 [46]	Y	PY	N	PY	Y	Y	N	PY	N	N	NA	NA	Y	N	NA	Y	CL
Zhu et al. 2014 [47]	Y	N	N	PY	N	Y	N	PY	Y	N	Y	Y	Y	Y	Y	Y	CL
Chen et al. 2015 [48]	Y	PY	N	PY	Y	Y	Y	Y	Y	N	N	Y	Y	Y	Y	Y	L
Li et al. 2015 [49]	Y	N	N	PY	N	Y	N	Y	Y	N	Y	N	N	N	Y	Y	CL
Liu et al. 2016 [50]	Y	N	N	PY	Y	Y	N	Y	Y	N	N	N	N	Y	N	Y	CL
Qin et al. 2016a [51]	Y	N	N	PY	Y	Y	N	PY	Y	N	N	N	N	N	N	Y	CL
Qin et al. 2016b [52]	Y	PY	N	Y	Y	Y	N	PY	Y	N	Y	N	N	Y	Y	Y	CL
Cai et al. 2017 [53]	Y	N	N	PY	Y	Y	N	PY	PY	N	N	Y	Y	N	N	Y	CL
Chang et al. 2017 [54]	Y	N	N	PY	Y	Y	N	Y	Y	N	Y	N	N	Y	Y	Y	CL
Anderson et al. 2018 [55]	Y	N	N	PY	N	N	N	PY	N	N	N	N	N	Y	N	N	CL
Franco et al. 2018 [56]	Y	Y	N	PY	Y	Y	Y	Y	Y	Y	Y	N	N	Y	N	Y	CL
Franco et al. 2019 [57]	Y	Y	N	Y	Y	Y	Y	Y	Y	Y	Y	N	N	Y	Y	Y	L
Liao et al. 2019 [58]	Y	N	N	PY	Y	Y	N	PY	Y	N	N	N	N	Y	Y	N	CL
Qin et al. 2019a [59]	Y	N	N	PY	Y	Y	N	PY	N	N	N	N	N	N	N	Y	CL
Qin et al. 2019b [60]	Y	N	N	PY	Y	Y	N	PY	N	N	N	N	N	N	N	Y	CL
Yuan et al. 2019 [61]	Y	N	N	PY	N	N	N	PY	Y	N	Y	N	N	N	N	Y	CL
Birowo et al. 2020 [62]	Y	PY	N	PY	Y	N	N	PY	Y	N	N	Y	Y	N	N	Y	CL
Huang et al. 2020 [63]	Y	N	N	PY	N	N	N	PY	N	N	N	N	N	N	Y	Y	CL
Li et al. 2020 [64]	Y	N	N	PY	N	N	N	PY	Y	N	N	N	N	N	N	Y	CL
Chen et al. 2021 [65]	Y	N	N	PY	Y	N	N	PY	N	N	N	N	N	N	Y	Y	CL
Kang et al. 2021 [66]	Y	N	N	PY	Y	Y	N	PY	Y	N	N	N	N	N	Y	Y	CL
Li et al. 2021 [67]	Y	N	N	PY	Y	Y	N	PY	N	N	N	N	N	Y	N	Y	CL
Mykoniatis et al. 2021 [68]	Y	PY	N	PY	Y	Y	Y	PY	Y	N	Y	N	Y	Y	Y	Y	M
Zhang et al. 2021a [69]	Y	N	N	PY	Y	Y	N	PY	PY	N	Y	N	N	N	N	Y	CL
Zhang et al. 2021b [70]	Y	PY	N	N	Y	Y	N	PY	Y	N	N	N	Y	N	Y	Y	CL
Kong et al. 2022 [71]	Y	Y	N	PY	Y	Y	N	PY	Y	N	Y	N	N	N	N	Y	CL
Lao et al. 2022 [72]	Y	PY	N	PY	Y	Y	N	PY	Y	N	Y	N	Y	N	Y	Y	L
Lok et al. 2022 [73]	Y	N	N	N	Y	Y	N	PY	Y	N	N	N	N	N	N	Y	CL
Andrey et al. 2022 [6]	Y	PY	Y	PY	Y	Y	N	PY	Y	N	N	N	N	N	Y	Y	CL
Qin et al. 2022a [7]	Y	PY	N	PY	Y	Y	N	PY	Y	N	Y	N	N	Y	N	Y	CL
Qin et al. 2022b [74]	Y	Y	N	PY	Y	Y	N	PY	Y	Y	Y	N	N	N	N	Y	CL
Zhao et al. 2022 [75]	N	N	N	PY	Y	Y	N	N	N	N	NA	NA	Y	N	NA	Y	CL

*Y* yes, *PY* partial yes, *N* no, *NA* not applicable, *CL* critically low, *L* low, *M* moderate

### Univariable analysis

We identified the association between each potential factor and methodological quality by Chi-square tests or Fisher’s exact tests. The methodological quality of SRs/MAs differed significantly in the continent (*P* = 0.027) and preregistration (*P* = 0.004). However, no significant differences were observed in publication year (*P* = 1.000), PRISMA (*P* = 0.589), funding support (*P* = 0.791), whether CDSR (*P* = 0.087), RCT enrollment (*P* = 0.590), and meta-analysis (*P* = 0.431). The detailed results are displayed in Table 3.

**Table 3:** Univariable analysis of potential factors affecting the methodologic quality of SRs/MAs of CP/CPPS (*N* = 45)

Characteristics	N (%) (*N* = 45)	Total (*N* = 45)			*P* value
		M (*N* = 2)	L (*N* = 3)	CL (*N* = 40)	
Publication year					1.000
Before 2010	7 (15.6)	0 (0.0)	0 (0.0)	7 (100.0)	
After 2010	38 (84.4)	2 (5.3)	3 (7.9)	33 (86.8)	
Continent					0.027
Asia	29 (64.4)	0 (0.0)	2 (6.9)	27 (93.1)	
Europe	6 (13.4)	2 (33.3)	0 (0.0)	4 (66.7)	
North America	8 (17.8)	0 (0.0)	0 (0.0)	8 (100.0)	
South America	2 (4.4)	0 (0.0)	1 (50.0)	1 (50.0)	
PRISMA					0.589
No	15 (33.3)	1 (6.7)	0 (0.0)	14 (93.3)	
Yes	30 (66.7)	1 (3.3)	3 (10.0)	26 (86.7)	
Preregistration					0.004
No	29 (64.4)	0 (0.0)	0 (0.0)	29 (100.0)	
Yes	16 (35.6)	2 (12.5)	3 (18.8)	11 (68.8)	
Funding support					0.791
No	21 (46.7)	1 (4.8)	2 (9.5)	18 (85.7)	
Yes	24 (53.3)	1 (4.2)	1 (4.2)	22 (91.6)	
RCT enrollment					0.590
non-RCTs	5 (11.1)	0 (0.0)	1 (20.0)	4 (80.0)	
RCTs	29 (64.4)	2 (6.9)	2 (6.9)	25 (86.2)	
RCTs and non-RCTs	11 (24.4)	0 (0.0)	0 (0.0)	11 (100.0)	
Whether CDSR					0.087
non-CDSR	40 (88.9)	1 (2.5)	2 (5.0)	37 (92.5)	
CDSR	5 (11.1)	1 (20.0)	1 (20.0)	3 (60.0)	
Meta-analysis					0.431
Without	9 (20.0)	1 (11.1)	0 (0.0)	8 (88.9)	
With	36 (80.0)	1 (2.8)	3 (8.3)	32 (88.9)	

Percentages may not sum to 100 due to rounding of values

*CL* critically low, *L* low, *M* moderate

### Multivariate analysis

No significant factors related to the methodological quality of systematic reviews were observed in multivariate analysis. SRs/MAs from Asia were similar to those from Europe (coefficient, 0.481; 95% CI, -0.040 to 1.002; *P* = 0.069), North America (coefficient, -0.064; 95% CI, -0.557 to 0.429; *P* = 0.793) and South America (coefficient, -0.299; 95% CI, -1.315 to 0.718; *P* = 0.554). The methodological quality of SRs/MAs that did not include RCTs was similar to those that included only RCTs (coefficient, -0.176; 95% CI, -0.624 to 0.272; *P* = 0.430), and those that included RCTs and non-RCTs (coefficient, -0.272; 95% CI, -0.808 to 0.264; *P* = 0.309). Furthermore, no significant differences of the methodological quality of SRs/MAs were observed in the publication year (coefficient, 0.333; 95% CI, -0.159 to 0.826; *P* = 0.117), PRISMA (coefficient, -0.105; 95% CI, -0.510 to 0.300; *P* = 0.602), preregistration (coefficient, 0.231; 95% CI, -0.138 to 0.600; *P* = 0.212), funding support (coefficient, -0.059; 95% CI, -0.416 to 0.297; *P* = 0.737), whether CDSR (coefficient, 0.532; 95% CI, -0.193 to 1.257; *P* = 0.145), and meta-analysis (coefficient, 0.041; 95% CI, -0.518 to 0.600; *P* = 0.882; Table 4).

**Table 4:** Multiple linear regression analysis of potential factors affecting the methodologic quality of SRs/MAs of CP/CPPS (*N* = 45)

Characteristics	Coefficients	95% CI	*P* value
Publication year (Before 2010)			
After 2010	0.333	-0.159, 0.826	0.177
Continent (Asia)			
Europe	0.481	-0.040, 1.002	0.069
North America	-0.064	-0.557, 0.429	0.793
South America	-0.299	-1.315, 0.718	0.554
PISMA (No)			
Yes	-0.105	-0.510, 0.300	0.602
Preregistration (No)			
Yes	0.231	-0.138, 0.600	0.212
Funding support (No)			
Yes	-0.059	-0.416, 0.297	0.737
RCT enrollment (non-RCTs)			
RCTs and non-RCTs	-0.272	-0.808, 0.264	0.309
RCTs	-0.176	-0.624, 0.272	0.430
Whether CDSR (non-CDSR)			
CDSR	0.532	-0.193, 1.257	0.145
Meta-analysis (Without)			
With	0.041	-0.518, 0.600	0.882

### Subgroup analysis

Considering that AMSTAR2 was developed based on interventional SRs/MAs, we conducted a subgroup analysis of the 38 included interventional SRs/MAs. Univariate analysis showed that the methodological quality of interventional SRs/MAs of CP/CPPS was significantly associated with the affiliated continent (*P* = 0.017) and preregistration (*P* = 0.014; Table 5). However, in multivariate analysis, only the continent remained significant. Specifically, SRs/MAs from Asia had a significant difference in methodological quality when compared with SRs/MAs from Europe (coefficient, 0.652; 95% CI, 0.046 to 1.258; *P* = 0.036), but were similar to those from North America (coefficient, -0.053; 95% CI, -0.639 to 0.532; *P* = 0.853) and South America (coefficient, -0.094; 95% CI, -1.233 to 1.045; *P* = 0.867; Table 6).

**Table 5:** Univariable analysis of potential factors affecting the methodologic quality of interventional SRs/MAs of CP/CPPS (*N* = 38)

Characteristics	N (%) (*N* = 38)	Total (*N* = 38)			*P* value
		M (*N* = 2)	L (*N* = 2)	CL (*N* = 34)	
Publication year					1.000
Before 2010	7 (18.4)	0 (0.0)	0 (0.0)	7 (100.0)	
After 2010	31 (81.6)	2 (6.5)	2 (6.5)	27 (87.1)	
Continent					0.017
Asia	23 (60.5)	0 (0.0)	1 (4.3)	22 (95.7)	
Europe	5 (13.2)	2 (40.0)	0 (0.0)	3 (60.0)	
North America	8 (21.1)	0 (0.0)	0 (0.0)	8 (100.0)	
South America	2 (5.3)	0 (0.0)	1 (50.0)	1 (50.0)	
PRISMA					0.773
No	14 (36.8)	1 (7.1)	0 (0.0)	13 (92.9)	
Yes	24 (63.2)	1 (4.2)	2 (8.3)	21 (87.5)	
Preregistration					0.014
No	24 (63.2)	0 (0.0)	0 (0.0)	24 (100.0)	
Yes	14 (36.8)	2 (14.3)	2 (14.3)	10 (71.4)	
Funding support					1.000
No	18 (47.4)	1 (5.6)	1 (5.6)	16 (88.9)	
Yes	20 (52.6)	1 (5.0)	1 (5.0)	18 (90.0)	
RCT enrollment					1.000
non-RCTs	1 (2.6)	0 (0.0)	0 (0.0)	1 (100.0)	
RCTs	29 (76.3)	2 (6.9)	2 (6.9)	25 (86.2)	
RCTs and non-RCTs	8 (21.1)	0 (0.0)	0 (0.0)	8 (100.0)	
Whether CDSR					0.076
non-CDSR	33 (86.8)	1 (3.0)	1 (3.0)	31 (93.9)	
CDSR	5 (13.2)	1 (20.0)	1 (20.0)	3 (60.0)	
Meta-analysis					0.574
Without	7 (18.4)	1 (14.3)	0 (0.0)	6 (85.7)	
With	31 (81.6)	1 (3.2)	2 (6.5)	28 (90.3)	

Percentages may not sum to 100 due to rounding of values

*CL* critically low, *L* low, *M* moderate

**Table 6:** Multiple linear regression analysis of potential factors affecting the methodologic quality of interventional SRs/MAs of CP/CPPS (*N* = 38)

Characteristics	Coefficients	95% CI	*P* value
Publication year (Before 2010)			
After 2010	0.398	-0.149, 0.944	0.147
Continent (Asia)			
Europe	0.652	0.046, 1.258	0.036
North America	-0.053	-0.639, 0.532	0.853
South America	-0.094	-1.233, 1.045	0.867
PRISMA (No)			
Yes	-0.134	-0.606, 0.338	0.564
Preregistration (No)			
Yes	0.194	-0.237, 0.625	0.362
Funding support (No)			
Yes	0.029	-0.384, 0.442	0.886
RCT enrollment (non-RCTs)			
RCTs and non-RCTs	-0.405	-1.470, 0.660	0.441
RCTs	-0.322	-1.424, 0.781	0.554
Whether CDSR (non-CDSR)			
CDSR	0.425	-0.422, 1.272	0.312
Meta-analysis (Without)			
With	-0.020	-0.817, 0.776	0.958

### Sensitivity analysis

As AMSTAR2 was developed in 2017, we performed a sensitivity analysis to explore the impact of the release of AMSTAR2 on the methodological quality of SRs/MAs of CP/CPPS. Thus, the publication years were changed to another two categories, that was before 2018 and after 2018 (including 2018). We found no substantial change in the results of both main and subgroup analysis (Additional files 6 and 7).

## Discussion

In this study, we assessed for the first time the methodological quality of the SRs/MAs of CP/CPPS. In general, the methodological quality of SRs/MAs of CP/CPPS was mostly unsatisfactory.

Although SRs/MAs are considered one of the highest levels of evidence, their quality varies considerably which affects their clinical applicability. Sun et al. reported that the quality of SRs/MAs in traditional Chinese medicine for ischemic stroke is poor, which led to a lack of timely access to valid information for clinicians [76]. In our study, a general lack of methodological rigor was noted in SRs/MAs of CP/CPPS, which might explain why there was no standardized management strategy for CP/CPPS until today to some extent. Therefore, the methodological quality of SRs/MAs of CP/CPPS should be improved. Firstly, to ensure standardization of the study implementation process and improve the openness, transparency, and reproducibility of the evidence, researchers should register or publish their study protocol in advance, which could avoid unnecessary duplication, reduce finite resources waste, and encourage cooperation [77]. Secondly, RCTs and observational studies often have complementary roles [28], and SRs/MAs might give an incomplete summary when only RCTs are included [17]. So, we strongly recommend that authors of SRs/MAs explain their selection of the study designs for inclusion. Thirdly, search strategies for SRs/MAs must be comprehensive and sensitive to ensure the inclusion of all relevant primary research [78]. However, relevant gray literature was often overlooked in most included SRs/MAs. Therefore, not only comprehensive search strategy should be used in the literature search process, but also gray literature (such as conference papers, and academic dissertations) should be searched manually. Fourthly, the lack of a list of excluded literature and the reasons often indicates less transparency in reporting [78]. Thus, a list of exclusions and reasons should be provided to further improve the rigor of the literature selection process in future studies. Fifthly, industry-funded studies are more likely to reach conclusions that benefit the industry [79, 80]. The AMSTAR2 tool added a review of the funding source for included studies in reviews, but funding sources and conflicts of interest were seldom reported in included SRs, which should be improved in the future. Sixthly, when significant heterogeneity exists, in addition to correctly applying the random or fixed effect model, researchers need to perform subgroup analysis or meta-regression to analyze the causes of heterogeneity and explain its impact on research results. Finally, while adhering to the relevant guidelines to minimize bias, researchers should be aware that it is not enough to assess the risk of bias, but must consider its impact on the results of the review.

Next, we explored potential factors affecting the methodological quality of SRs/MAs of CP/CPPS. Univariate analysis showed that the methodological quality of SRs/MAs of CP/CPPS is significantly associated with continent and preregistration. Considering that the vast majority of the SRs/MAs included in the study were assessed as critically low quality, the probability of estimation of this level of quality is always close to 1 and does not require fitting an ordered regression model. Therefore, we employed the multivariate linear regression model with the overall score as the dependent variable. However, no factors including continent and preregistration were found significantly associated with the methodological quality of SRs/MAs of CP/CPPS in the multivariate analysis. In fact, the study region was not always classified consistently in published literature. A study found that there was a significant difference in reporting and methodologic qualities in published surgical MAs from Asia and non-Asia [27]. Differently, some studies did not find the impact of the study region on the methodological quality of the MAs, in which the study regions were divided more detailedly [81, 82]. The impact of the study region on methodological quality of SRs/MAs of CP/CPPS should be further explored in the future. Similarly, unlike the results of univariate analysis, preregistration was also found not to correlate with the methodological quality in multivariate analysis. This may be due to the small sample size, which needs to be further expanded in the future to verify the effect of registration on the quality of SRs/MAs of CP/CPPS. Preregistration can ensure a more standardized study process and help to reduce the selective outcome-reporting bias [83]. Previous studies have shown that preregistration was independently associated with superior methodological quality and contributes to the methodological quality of SRs [27, 84]. Therefore, preregistration may be an effective measure to improve the methodological quality of SRs and should be taken into account by researchers.

Previous studies have illustrated that the quality of SRs/MAs tends to improve gradually over time [15, 78, 81, 85]. However, although the vast majority of SRs/MAs (n = 38, 84.4%) of CP/CPPS were published after 2010, we found that the methodological quality of SRs/MAs of CP/CPPS did not significantly improve after 2010. The PRISMA statement was developed to promote transparent and complete reporting of systematic reviews [86]. Some researchers found a positive correlation between AMSTAR and PRISMA scores in SRs [30, 87]. However, we did not observe the impact of adhering to PRISMA on the methodological quality of SRs/MAs of CP/CPPS in this study. Additionally, some studies found that SRs/MAs with conflicts of interest tend to have lower methodological quality and reach favorable conclusions than those without financial conflicts of interest [88, 89]. Ghozy et al. found that financial support does not significantly affect the overall quality of SRs which was consistent with our results, and they found that funded studies tend to include more RCTs and report conflicts of interest more frequently than non-funded ones [90]. The reliability of the conclusions of SRs also depends on the quality of the initial studies included. To compare the advantages and disadvantages of various interventions, the best form of evidence is a rigorously designed RCT with an adequate sample size [91]. RCTs are known to have the highest quality of evidence of all study types, which makes them the gold standard for evidence synthesis [28]. However, we did not find the influence of RCT enrollment on the methodological quality of SRs/MAs of CP/CPPS in this study which should be further validated in the future. Some studies reported that non-CDSR often had lower statistical precision despite reporting larger effect sizes than CDSR, which may be due to the more standardized methodology and more transparent reporting of Cochrane reviews [23, 92]. However, we did not observe such a difference in our study. Meta-analysis inclusion in SRs was regarded as a potential influencing factor in many studies, and those studies found that SRs with a meta-analysis have a higher AMSTAR2 score [32, 85]. As a useful tool for summarizing research evidence, meta-analysis might not be always applicable because of clinical or statistical heterogeneity. Our results did not show the association between the presence of a meta-analysis and methodological quality of SRs of CP/CPPS. It might be that authors often did not conduct meta-analysis due to significant heterogeneity while such a situation might not affect the final quality of the produced SRs.

Notably, AMSTAR2 was initially used only for SRs/MAs that include healthcare interventions. Nevertheless, in recent years, a considerable number of studies have tried to use AMSTAR2 for non-interventional SRs/Mas [15, 93], so we included both intervention and non-intervention SRs/MAs in our initial analyses. Subsequently, we conducted a subgroup analysis after excluding non-interventional SRs/MAs, and the univariate analysis was consistent with the results of the initial analyses. Differently, in the multivariate analysis of intervention SRs, the continent remained significantly associated with the methodological quality of SRs/MAs of CP/CPPS. Particularly, there were significant differences in methodological quality between SRs/MAs from Asia and Europe. The difference between the main analysis and the subgroup analysis in the multivariate analysis might be due to a more stringent classification of the types of SRs which implied lower heterogeneity. However, due to the small sample size of this study and the large number of independent variables included in the regression analysis, these results should be further confirmed. Subsequently, to further explore whether the methodological quality of the SRs has improved after the release of AMSTAR, we used 2018 as the cut-off year in the sensitivity analysis. We reconducted the regression analyses and found that the results of the analysis did not change substantially. This validated the robustness of the results of the main and subgroup analysis to some extent.

### Study limitations

This study has several limitations. First, although AMSTAR2 was adopted in this study for the methodology evaluation of CP/CPPS-related SRs, there is no gold standard for assessing the quality of SRs. However, AMSTAR2 is one of the most widely used tools for evaluating the methodology quality of SRs. Second, only SRs/MAs written in English or Chinese were included. Thus, publication bias might exist. Third, we used VIF to test whether there is multicollinearity between factors. Although we did not find multicollinearity, there may be some correlation between individual variables. Fourth, a small sample of study were considered in this study while a relatively large number of influencing variables were adopted in the analysis, which might affect the stability of the results and require further verification in the future. Fifth, due to limitations in the design of cross-sectional studies, our findings may not apply to other disease areas of medicine. Finally and noteworthyly, there were some differences between the registered protocol and the manuscript. For example, there were not any restrictions to the publication language in the protocol while only studies published in Chinese or English were included in the final manuscript. No publication language restriction guaranteed the comprehensiveness of the conducting literature retrieval. However, considering the accuracy of translation and data extraction, and few publications in non-Chinese or non-English after retrieval, only the Chinese and English documents were included. Additionally, as stated in the protocol, we planned to use the PRISMA checklist, the AMSTAR2 tool, the ROBIS tool, and the GRADE system to assess the quality of CP/CPPS-related SRs comprehensively through various aspects such as quality of reporting, methodological quality, risk of bias, and grading of evidence [94–98]. Also, we intended to assess the clinical efficacy of different interventions in SRs. However, this is a big project, especially when there is a lot of clinical efficacy data to evaluate and grade. Thus, we only attempted to explore the methodological quality and influencing factors in this preliminary study utilizing a widely adopted AMSTAR2 tool [17]. Although it seemed to be part of the preconceived study, this study is an exploratory work while the influencing factors we explored might be certainly enlightening. Additionally, it might provide some reference for our follow-up research to comprehensively evaluate the study quality and evidence grade about CP/CPPS-related SRs based on the registered protocol. In short, possible associations between methodological rigor and review characteristics, as well as comprehensive methodological features and evidence grading of CP/CPPS-related SRs should be further explored in the future.

## Conclusions

The methodological quality of SRs/MAs of CP/CPPS was suboptimal, and most were rated as low and critically low. In addition, this study identified domains where the methodological quality of SRs/MAs of CP/CPPS could be improved. Researchers should strictly adhere to the AMSTAR2 items to improve the methodological quality of SRs/MAs in the future. Although none of the investigated factors showed the association with the methodological quality of all types of SRs/MAs of CP/CPPS, the continent was associated with the methodological quality of a subgroup of interventional SRs/MAs of CP/CPPS.

## Supplementary Information

**Additional file 1.** 

**Additional file 2.** 

**Additional file 3.** 

**Additional file 4.** 

**Additional file 5.** 

**Additional file 6.** 

**Additional file 7.** 

## Data Availability

The data analyzed in this article can all be found within the article text, tables, figures, and supplementary material.

## Abbreviations

SRs/Mas: Systematic reviews/meta-analyses
RCT: Randomised controlled trial
CCT: Controlled clinical trial
NRSI: Non-randomised studies of the effects of interventions
CP/CPPS: Chronic prostatitis/chronic pelvic pain syndrome
SD: Sexual dysfunction
PRISMA: Preferred Reporting Items for systematic review and meta-analysis
AMSTAR: A Measurement Tool to Assess systematic Reviews
VIFs: Variance inflation factors
CDSR: Cochrane Database of Systematic Reviews

## References

[1] Krieger JN, Lee SW, Jeon J, Cheah PY, Liong ML, Riley DE. Epidemiology of prostatitis. Int J Antimicrob Agents. 2008; 31(Suppl 1):S85–90. 10.1016/j.ijantimicag.2007.08.028 PMC2292121 18164907

[2] Rees J, Abrahams M, Doble A, Cooper A. Diagnosis and treatment of chronic bacterial prostatitis and chronic prostatitis/chronic pelvic pain syndrome: a consensus guideline. BJU Int. 2015;116(4):509–25. 25711488 10.1111/bju.13101 PMC5008168

[3] Zhang J, Liang C, Shang X, Li H. Chronic prostatitis/Chronic pelvic pain syndrome: a disease or symptom? Current perspectives on diagnosis, treatment, and prognosis. Am J Mens Health. 2020;14(1):1557988320903200. 32005088 10.1177/1557988320903200 PMC7256330

[4] Krieger JN, Nyberg L Jr, Nickel JC. NIH consensus definition and classification of prostatitis. JAMA. 1999;282(3):236–7. 10422990 10.1001/jama.282.3.236

[5] Pena VN, Engel N, Gabrielson AT, Rabinowitz MJ, Herati AS. Diagnostic and management strategies for patients with chronic prostatitis and chronic pelvic pain syndrome. Drugs Aging. 2021;38(10):845–86. 34586623 10.1007/s40266-021-00890-2

[6] Morozov A, Bazarkin A, Babaevskaya D, Taratkin M, Kozlov V, Suvorov A, et al. A systematic review and meta-analysis of placebo effect in clinical trials on chronic prostatitis/chronic pelvic pain syndrome. Prostate. 2022;82(6):633–56. 35133667 10.1002/pros.24311

[7] Qin Z, Guo J, Chen H, Wu J. Acupuncture for chronic prostatitis/chronic pelvic pain syndrome: a GRADE-assessed systematic review and meta-analysis. European urology open science. 2022;46:55–67. 36506258 10.1016/j.euros.2022.10.005 PMC9732484

[8] Maeda K, Shigemura K, Fujisawa M. A review of current treatments for chronic prostatitis/chronic pelvic pain syndrome under the UPOINTS system. Int J Urol. 2023;30(5):431–6. 10.1111/iju.15149 36788717

[9] Nickel JC. Chronic prostatitis/chronic pelvic pain syndrome: it is time to change our management and research strategy. BJU Int. 2020;125(4):479–80. 32250053 10.1111/bju.15036

[10] Cai T, Alidjanov J, Palagin I, Medina-Polo J, Nickel JC, Wagenlehner FME. Chronic prostatitis/chronic pelvic pain syndrome (CP/CPPS): look to the future. Prostate cancer and prostatic diseases. 2023. 10.1038/s41391-023-00645-7 36631538

[11] Horwitz RI, Hayes-Conroy A, Caricchio R, Singer BH. From evidence based medicine to medicine based evidence. Am J Med. 2017;130(11):1246–50. 28711551 10.1016/j.amjmed.2017.06.012

[12] Zhao M, Gao QH, Liu SJ, Deng YJ, Wang H, Yu WX, et al. Quality assessment and relevant clinical impact of randomized controlled trials on chronic prostatitis/chronic pelvic pain syndrome. BMC Urol. 2022;22(1):122. 35941610 10.1186/s12894-022-01078-5 PMC9361679

[13] Djulbegovic B, Guyatt GH. Progress in evidence-based medicine: a quarter century on. Lancet (London, England). 2017;390(10092):415–23. 28215660 10.1016/S0140-6736(16)31592-6

[14] Young D. Policymakers, experts review evidence-based medicine. Am J Health Syst Pharm. 2005;62(4):342–3. 15745880 10.1093/ajhp/62.4.0342

[15] Hammel C, Pandis N, Pieper D, Faggion CM Jr. Methodological assessment of systematic reviews of in-vitro dental studies. BMC Med Res Methodol. 2022;22(1):110. 35413840 10.1186/s12874-022-01575-z PMC9006561

[16] Dong C, Shi H, Liu P, Si G, Yan Z. A critical overview of systematic reviews and meta-analyses of light therapy for non-seasonal depression. Psychiatry Res. 2022;314: 114686. 35753223 10.1016/j.psychres.2022.114686

[17] Shea BJ, Reeves BC, Wells G, Thuku M, Hamel C, Moran J, et al. AMSTAR 2: a critical appraisal tool for systematic reviews that include randomised or non-randomised studies of healthcare interventions, or both. BMJ. 2017;358: j4008. 28935701 10.1136/bmj.j4008 PMC5833365

[18] Shea BJ, Grimshaw JM, Wells GA, Boers M, Andersson N, Hamel C, et al. Development of AMSTAR: a measurement tool to assess the methodological quality of systematic reviews. BMC Med Res Methodol. 2007;7:10. 17302989 10.1186/1471-2288-7-10 PMC1810543

[19] Whiting P, Savovic J, Higgins JP, Caldwell DM, Reeves BC, Shea B, et al. ROBIS: a new tool to assess risk of bias in systematic reviews was developed. J Clin Epidemiol. 2016;69:225–34. 26092286 10.1016/j.jclinepi.2015.06.005 PMC4687950

[20] Booth A, Clarke M, Dooley G, Ghersi D, Moher D, Petticrew M, et al. The nuts and bolts of PROSPERO: an international prospective register of systematic reviews. Syst Rev. 2012;1:2. 22587842 10.1186/2046-4053-1-2 PMC3348673

[21] Page MJ, Shamseer L, Tricco AC. Registration of systematic reviews in PROSPERO: 30,000 records and counting. Syst Rev. 2018;7(1):32. 29463298 10.1186/s13643-018-0699-4 PMC5819709

[22] Yanan Wang YL, Shuai Liu, Xin Guan, Xiaolong Li, Zewen Li, Zhilong Dong. Methodological quality and clinical outcomes of systematic reviews on chronic prostatitis/chronic pelvic pain syndrome: an overview: PROSPERO 2022 CRD42022343957 Available from: https://www.crd.york.ac.uk/prospero/display_record.php?ID=CRD42022343957; 2022.

[23] Moseley AM, Elkins MR, Herbert RD, Maher CG, Sherrington C. Cochrane reviews used more rigorous methods than non-Cochrane reviews: survey of systematic reviews in physiotherapy. J Clin Epidemiol. 2009;62(10):1021–30. 19282144 10.1016/j.jclinepi.2008.09.018

[24] Aromataris E, Fernandez R, Godfrey CM, Holly C, Khalil H, Tungpunkom P. Summarizing systematic reviews: methodological development, conduct and reporting of an umbrella review approach. Int J Evid Based Healthc. 2015;13(3):132–40. 26360830 10.1097/XEB.0000000000000055

[25] Cumpston M, Li T, Page MJ, Chandler J, Welch VA, Higgins JP, et al. Updated guidance for trusted systematic reviews: a new edition of the Cochrane Handbook for Systematic Reviews of Interventions. Cochrane Database Syst Rev. 2019;10(10):ED000142. 10.1002/14651858.ED000142 PMC10284251 31643080

[26] Liberati A, Altman DG, Tetzlaff J, Mulrow C, Gøtzsche PC, Ioannidis JPA, et al. The PRISMA statement for reporting systematic reviews and meta-analyses of studies that evaluate health care interventions: explanation and elaboration. PLoS Med. 2009;6(7): e1000100. 19621070 10.1371/journal.pmed.1000100 PMC2707010

[27] Zhang H, Han J, Zhu YB, Lau WY, Schwartz ME, Xie GQ, et al. Reporting and methodological qualities of published surgical meta-analyses. J Clin Epidemiol. 2016;70:4–16. 26117439 10.1016/j.jclinepi.2015.06.009

[28] Barton S. Which clinical studies provide the best evidence? The best RCT still trumps the best observational study. BMJ (Clinical research ed). 2000;321(7256):255–6. 10915111 10.1136/bmj.321.7256.255 PMC1118259

[29] Sideri S, Papageorgiou SN, Eliades T. Registration in the international prospective register of systematic reviews (PROSPERO) of systematic review protocols was associated with increased review quality. J Clin Epidemiol. 2018;100:103–10. 29339215 10.1016/j.jclinepi.2018.01.003

[30] Ross A, Rankin J, Beaman J, Murray K, Sinnett P, Riddle R, et al. Methodological quality of systematic reviews referenced in clinical practice guidelines for the treatment of opioid use disorder. PLoS One. 2017;12(8). 10.1371/journal.pone.0181927 PMC5542448 28771633

[31] Pieper D, Hellbrecht I, Zhao L, Baur C, Pick G, Schneider S, et al. Impact of industry sponsorship on the quality of systematic reviews of vaccines: a cross-sectional analysis of studies published from 2016 to 2019. Syst Rev. 2022;11(1):174. 35996186 10.1186/s13643-022-02051-x PMC9395849

[32] Sun X, Wang D, Wang M, Li H, Liu B. The reporting and methodological quality of systematic reviews and meta-analyses of nursing interventions for chronic obstructive pulmonary disease - A systematic review. Nurs Open. 2021;8(3):1489–500. 33465288 10.1002/nop2.767 PMC8046131

[33] McNaughton C, Mac Donald R, Wilt T. Interventions for chronic abacterial prostatitis. Cochrane Database Syst Rev. 2001;1999(1):Cd002080. 10.1002/14651858.CD002080 PMC7017856 11279750

[34] McNaughton Collins M, MacDonald R, Wilt TJ. Diagnosis and treatment of chronic abacterial prostatitis: a systematic review. Ann Intern Med. 2000;133(5):367–81. 10979882 10.7326/0003-4819-133-5-200009050-00013

[35] McNaughton CO, Wilt T. Allopurinol for chronic prostatitis. Cochrane Database Syst Rev. 2002(4):Cd001041. 10.1002/14651858.CD001041 12519549

[36] Yang G, Wei Q, Li H, Yang Y, Zhang S, Dong Q. The effect of alpha-adrenergic antagonists in chronic prostatitis/chronic pelvic pain syndrome: a meta-analysis of randomized controlled trials. J Androl. 2006;27(6):847–52. 16870951 10.2164/jandrol.106.000661

[37] Lee SW, Liong ML, Yuen KH, Liong YV, Krieger JN. Chronic prostatitis/chronic pelvic pain syndrome: role of alpha blocker therapy. Urol Int. 2007;78(2):97–105. 17293646 10.1159/000098064

[38] Mishra VC, Browne J, Emberton M. Role of alpha-blockers in type III prostatitis: a systematic review of the literature. J Urol. 2007;177(1):25–30. 17161995 10.1016/j.juro.2006.08.090

[39] Yang GM, Zhao XK, Kou Y. Management of chronic prostatitis/chronic pelvic pain syndrome (CP/CPPS): a systematic review and meta-analysis of randomized controlled trials (RCTs). Chinese J Androl. 2008;04:20–6.

[40] Anothaisintawee T, Attia J, Nickel JC, Thammakraisorn S, Numthavaj P, McEvoy M, et al. Management of chronic prostatitis/chronic pelvic pain syndrome: a systematic review and network meta-analysis. JAMA. 2011;305(1):78–86. 21205969 10.1001/jama.2010.1913

[41] Aboumarzouk OM, Nelson RL. Pregabalin for chronic prostatitis. Cochrane Database Syst Rev. 2012(8):Cd009063. 10.1002/14651858.CD009063.pub2 PMC11994983 22895982

[42] Cohen JM, Fagin AP, Hariton E, Niska JR, Pierce MW, Kuriyama A, et al. Therapeutic intervention for chronic prostatitis/chronic pelvic pain syndrome (CP/CPPS): a systematic review and meta-analysis. PLoS ONE. 2012;7(8): e41941. 22870266 10.1371/journal.pone.0041941 PMC3411608

[43] Thakkinstian A, Attia J, Anothaisintawee T, Nickel JC. α-blockers, antibiotics and anti-inflammatories have a role in the management of chronic prostatitis/chronic pelvic pain syndrome. BJU Int. 2012;110(7):1014–22. 22471591 10.1111/j.1464-410X.2012.11088.x

[44] Herati AS, Moldwin RM. Alternative therapies in the management of chronic prostatitis/chronic pelvic pain syndrome. World J Urol. 2013;31(4):761–6. 23740129 10.1007/s00345-013-1097-0

[45] Fu W, Zhou Z, Liu S, Li Q, Yao J, Li W, et al. The effect of chronic prostatitis/chronic pelvic pain syndrome (CP/CPPS) on semen parameters in human males: a systematic review and meta-analysis. PLoS One. 2014;9(4). 24743301 10.1371/journal.pone.0094991 PMC3990624

[46] Riegel B, Bruenahl CA, Ahyai S, Bingel U, Fisch M, Löwe B. Assessing psychological factors, social aspects and psychiatric co-morbidity associated with Chronic Prostatitis/Chronic Pelvic Pain Syndrome (CP/CPPS) in men – a systematic review. J Psychosom Res. 2014;77(5):333–50. 25300538 10.1016/j.jpsychores.2014.09.012

[47] Zhu Y, Wang C, Pang X, Li F, Chen W, Tan W. Antibiotics are not beneficial in the management of category III prostatitis: a meta analysis. Urol J. 2014;11(2):1377–85. 24807747

[48] Chen X, Zhou Z, Qiu X, Wang B, Dai J. The effect of chronic Prostatitis/Chronic Pelvic Pain Syndrome (CP/CPPS) on erectile function: a systematic review and meta-analysis. PLoS ONE. 2015;10(10). 26509575 10.1371/journal.pone.0141447 PMC4625019

[49] Li HJ, Kang DY. Prevalence of sexual dysfunction in men with chronic prostatitis/chronic pelvic pain syndrome: a meta-analysis. World J Urol. 2016;34(7):1009–17. 26546073 10.1007/s00345-015-1720-3 PMC4921105

[50] Liu BP, Wang YT, Chen SD. Effect of acupuncture on clinical symptoms and laboratory indicators for chronic prostatitis/chronic pelvic pain syndrome: a systematic review and meta-analysis. Int Urol Nephrol. 2016;48(12):1977–91. 27590134 10.1007/s11255-016-1403-z

[51] Qin Z, Wu J, Tian J, Zhou J, Liu Y, Liu Z. Network Meta-Analysis of the Efficacy of Acupuncture, Alpha-blockers and Antibiotics on Chronic Prostatitis/Chronic Pelvic Pain Syndrome. Sci Rep. 2016;6:35737. 27759111 10.1038/srep35737 PMC5069632

[52] Qin Z, Wu J, Zhou J, Liu Z. Systematic Review of Acupuncture for Chronic Prostatitis/Chronic Pelvic Pain Syndrome. Medicine. 2016;95(11): e3095. 26986148 10.1097/MD.0000000000003095 PMC4839929

[53] Cai T, Verze P, La Rocca R, Anceschi U, De Nunzio C, Mirone V. The role of flower pollen extract in managing patients affected by chronic prostatitis/chronic pelvic pain syndrome: a comprehensive analysis of all published clinical trials. BMC Urol. 2017;17(1):32. 28431537 10.1186/s12894-017-0223-5 PMC5401347

[54] Chang SC, Hsu CH, Hsu CK, Yang SS, Chang SJ. The efficacy of acupuncture in managing patients with chronic prostatitis/chronic pelvic pain syndrome: a systemic review and meta-analysis. Neurourol Urodyn. 2017;36(2):474–81. 26741647 10.1002/nau.22958

[55] Anderson RU, Wise D, Nathanson BH. Chronic Prostatitis and/or Chronic Pelvic Pain as a Psychoneuromuscular Disorder-A Meta-analysis. Urology. 2018;120:23–9. 30056195 10.1016/j.urology.2018.07.022

[56] Franco JV, Turk T, Jung JH, Xiao YT, Iakhno S, Garrote V, et al. Non-pharmacological interventions for treating chronic prostatitis/chronic pelvic pain syndrome. Cochrane Database Syst Rev. 2018;1(1):Cd012551. 10.1002/14651858.CD012551.pub2 PMC6491290 29372565

[57] Franco JV, Turk T, Jung JH, Xiao YT, Iakhno S, Tirapegui FI, et al. Pharmacological interventions for treating chronic prostatitis/chronic pelvic pain syndrome. Cochrane Database Syst Rev. 2019;10(10):Cd012552. 10.1002/14651858.CD012552.pub2 PMC6778620 31587256

[58] Liao B, Mou XX, Liu JB, Wu T, Cui S. [Extracorporeal shock wave therapy for chronic prostatitis / chronic pelvic pain syndrome: a meta-analysis]. Natl J Androl. 2019;25(10):914–22. 32233224

[59] Qin Z, Wu J, Xu C, Liu Z. Using meta-regression approach to explore the dose-response association between acupuncture sessions and acupuncture effects on chronic prostatitis/chronic pelvic pain syndrome. Ann Transl Med. 2019;7(6):116. 31032271 10.21037/atm.2018.11.45 PMC6465436

[60] Qin Z, Wu J, Xu C, Sang X, Li X, Huang G, et al. Long-term effects of acupuncture for chronic prostatitis/chronic pelvic pain syndrome: systematic review and single-arm meta-analyses. Ann Transl Med. 2019;7(6):113. 31032268 10.21037/atm.2018.06.44 PMC6465443

[61] Yuan P, Ma D, Zhang Y, Gao X, Liu Z, Li R, et al. Efficacy of low-intensity extracorporeal shock wave therapy for the treatment of chronic prostatitis/chronic pelvic pain syndrome: a systematic review and meta-analysis. Neurourol Urodyn. 2019;38(6):1457–66. 31037757 10.1002/nau.24017

[62] Birowo P, Rangganata E, Rasyid N, Atmoko W. Efficacy and safety of extracorporeal shockwave therapy for the treatment of chronic non-bacterial prostatitis: a systematic review and meta-analysis. PLoS One. 2020;15(12). 33370372 10.1371/journal.pone.0244295 PMC7769278

[63] Huang X, Qin Z, Cui H, Chen J, Liu T, Zhu Y, et al. Psychological factors and pain catastrophizing in men with chronic prostatitis/chronic pelvic pain syndrome (CP/CPPS): a meta-analysis. Transl Androl Urol. 2020;9(2):485–93. 32420154 10.21037/tau.2020.01.25 PMC7214995

[64] Li J, Dong L, Yan X, Liu X, Li Y, Yu X, et al. Is acupuncture another good choice for physicians in the treatment of chronic Prostatitis/Chronic Pelvic Pain Syndrome? Review of the latest literature. Pain Res Manage. 2020;2020:5921038. 10.1155/2020/5921038 PMC7085851 32256909

[65] Chen L, Bian Z, Chen J, Meng J, Zhang M, Liang C. Immunological alterations in patients with chronic prostatitis/chronic pelvic pain syndrome and experimental autoimmune prostatitis model: a systematic review and meta-analysis. Cytokine. 2021;141. 33550164 10.1016/j.cyto.2021.155440

[66] Kang Y, Song P, Cao D, Di X, Lu Y, Liu P, et al. The efficacy and safety of extracorporeal shockwave therapy versus acupuncture in the management of Chronic Prostatitis/Chronic Pelvic Pain Syndrome: evidence based on a network meta-analysis. Am J Mens Health. 2021;15(6):15579883211057998. 34911370 10.1177/15579883211057998 PMC8721709

[67] Li G, Man L. Low-intensity extracorporeal shock wave therapy for male chronic pelvic pain syndrome: a systematic review and meta-analysis. Transl Androl Urol. 2021;10(3):1202–11. 33850755 10.21037/tau-20-1423 PMC8039608

[68] Mykoniatis I, Pyrgidis N, Sokolakis I, Sountoulides P, Hatzichristodoulou G, Apostolidis A, et al. Low-intensity shockwave therapy for the management of chronic prostatitis/chronic pelvic pain syndrome: a systematic review and meta-analysis. BJU Int. 2021;128(2):144–52. 33434323 10.1111/bju.15335

[69] Zhang W, Fang Y, Shi M, Zhang M, Chen Y, Zhou T. Optimal acupoint and session of acupuncture for patients with chronic prostatitis/chronic pelvic pain syndrome: a meta-analysis. Transl Androl Urol. 2021;10(1):143–53. 33532304 10.21037/tau-20-913 PMC7844493

[70] Zhang Y, Ma H, Nan T, Li Y, Zheng W, Zhou Z, et al. Comparative efficacy of oral Chinese patent medicine for chronic Prostatitis/Chronic Pelvic Pain syndrome with sexual dysfunction: a Bayesian network meta-analysis of randomized controlled trials. Front Pharmacol. 2021;12. 34040523 10.3389/fphar.2021.649470 PMC8143435

[71] Kong X, Hu W, Dong Z, Tian J, Wang Y, Jin C, et al. The efficacy and safety of low-intensity extracorporeal shock wave treatment combined with or without medications in Chronic Prostatitis/Chronic Pelvic pain syndrome: a systematic review and meta-analysis. Prostate Cancer Prostat Dis. 2022. 10.1038/s41391-022-00571-0 35798855

[72] Lao Y, He L, Zhang P, Dong Z. Efficacy and safety of selective serotonin re-uptake inhibitors for chronic prostatitis/chronic pelvic pain syndrome: a systematic review and network meta-analysis. Asian J Surg. 2022;45(12):2810–2. 35739025 10.1016/j.asjsur.2022.06.047

[73] Lok W, Lin T, Cao D, Wei Q. Is Serenoa repens effective for the treatment of chronic prostatitis/chronic pelvic pain syndrome (CP/CPPS)? A systematic review and meta-analysis. Asian J Surg. 2022;45(9):1746–7. 35165019 10.1016/j.asjsur.2022.01.038

[74] Qin Z, Zhang C, Guo J, Kwong JSW, Li X, Pang R, et al. Oral pharmacological treatments for chronic prostatitis/chronic pelvic pain syndrome: a systematic review and network meta-analysis of randomised controlled trials. EClinicalMedicine. 2022;48. 35706494 10.1016/j.eclinm.2022.101457 PMC9125656

[75] Zhao Y, Lin J, Dong Y, Tian Z, Ye Y, Ma Z, et al. Neuroimaging studies of Chronic Prostatitis/Chronic pelvic pain syndrome. Pain Res Manage. 2022;2022:9448620. 10.1155/2022/9448620 PMC9095382 35573644

[76] Sun S, Zhao L, Zhou X, Liu X, Xie Z, Ren J, et al. Methodological, reporting, and evidence quality of systematic reviews of traditional Chinese medicine for ischemic stroke. Front Pharmacol. 2023;14:1047650. 36843924 10.3389/fphar.2023.1047650 PMC9947652

[77] Ioannidis JP, Greenland S, Hlatky MA, Khoury MJ, Macleod MR, Moher D, et al. Increasing value and reducing waste in research design, conduct, and analysis. Lancet (London, England). 2014;383(9912):166–75. 24411645 10.1016/S0140-6736(13)62227-8 PMC4697939

[78] Tumeh RA, Neto MS, Sales GD, Ferreira LM. Quality regarding the systematic reviews in breast plastic surgery. Aesthetic Plast Surg. 2023;47(2):559–67. 36781421 10.1007/s00266-023-03264-8

[79] Chartres N, Fabbri A, Bero LA. Association of industry sponsorship with outcomes of nutrition studies: a systematic review and meta-analysis. JAMA Intern Med. 2016;176(12):1769–77. 27802480 10.1001/jamainternmed.2016.6721

[80] Lexchin J, Bero LA, Djulbegovic B, Clark O. Pharmaceutical industry sponsorship and research outcome and quality: systematic review. BMJ (Clinical research ed). 2003;326(7400):1167–70. 12775614 10.1136/bmj.326.7400.1167 PMC156458

[81] Yan P, Yao L, Li H, Zhang M, Xun Y, Li M, et al. The methodological quality of robotic surgical meta-analyses needed to be improved: a cross-sectional study. J Clin Epidemiol. 2019;109:20–9. 30579979 10.1016/j.jclinepi.2018.12.013

[82] Tian J, Zhang J, Ge L, Yang K, Song F. The methodological and reporting quality of systematic reviews from China and the USA are similar. J Clin Epidemiol. 2017;85:50–8. 28063911 10.1016/j.jclinepi.2016.12.004

[83] Moher D, Liberati A, Tetzlaff J, Altman DG. Preferred reporting items for systematic reviews and meta-analyses: the PRISMA statement. BMJ (Clinical research ed). 2009;339: b2535. 19622551 10.1136/bmj.b2535 PMC2714657

[84] Ge L, Tian JH, Li YN, Pan JX, Li G, Wei D, et al. Association between prospective registration and overall reporting and methodological quality of systematic reviews: a meta-epidemiological study. J Clin Epidemiol. 2018;93:45–55. 29111471 10.1016/j.jclinepi.2017.10.012

[85] La Torre G, Bova R, Cocchiara RA, Sestili C, Tagliaferri A, Maggiacomo S, et al. What are the determinants of the quality of systematic reviews in the international journals of occupational medicine? A methodological study review of published literature. Int J Environ Res Public Health. 2023;20(2):1644. 10.3390/ijerph20021644 PMC9862101 36674398

[86] Page MJ, Moher D, Bossuyt PM, Boutron I, Hoffmann TC, Mulrow CD, et al. PRISMA 2020 explanation and elaboration: updated guidance and exemplars for reporting systematic reviews. BMJ (Clinical research ed). 2021;372: n160. 33781993 10.1136/bmj.n160 PMC8005925

[87] Yuan M, Wu J, Austin RE, Hofer SOP, Lista F, Ahmad J. Evaluating Breast Reconstruction Reviews Using A Measurement Tool to Assess Systematic Reviews (AMSTAR). Plast Reconstr Surg Glob Open. 2021;9(11): e3897. 34815919 10.1097/GOX.0000000000003897 PMC8604032

[88] Hansen C, Lundh A, Rasmussen K, Hróbjartsson A. Financial conflicts of interest in systematic reviews: associations with results, conclusions, and methodological quality. The Cochrane database of systematic reviews. 2019;8(8):Mr000047. 10.1002/14651858.MR000047.pub2 PMC7040976 31425611

[89] Lundh A, Lexchin J, Mintzes B, Schroll JB, Bero L. Industry sponsorship and research outcome. Cochrane Database Syst Rev. 2017;2(2):Mr000033. 10.1002/14651858.MR000033.pub3 PMC8132492 28207928

[90] Ghozy S, El-Qushayri AE, Gbreel MI, Farahat RA, Azzam AY, Elfil M, et al. The impact of funding on the quality and interpretation of systematic reviews of mechanical thrombectomy in stroke patients. Int Neuroradiology. 2022:15910199221145741. 10.1177/15910199221145741 PMC13294527 36852503

[91] Askie L, Offringa M. Systematic reviews and meta-analysis. Semin Fetal Neonatal Med. 2015;20(6):403–9. 26515266 10.1016/j.siny.2015.10.002

[92] Useem J, Brennan A, LaValley M, Vickery M, Ameli O, Reinen N, et al. Systematic differences between cochrane and non-cochrane meta-analyses on the same topic: a matched pair analysis. PLoS One. 2015;10(12). 26671213 10.1371/journal.pone.0144980 PMC4686011

[93] Luo J, Chen Z, Liu D, Li H, He S, Zeng L, et al. Methodological quality and reporting quality of COVID-19 living systematic review: a cross-sectional study. BMC Med Res Methodol. 2023;23(1):175. 37525117 10.1186/s12874-023-01980-y PMC10388517

[94] Gomez-Garcia F, Ruano J, Gay-Mimbrera J, Aguilar-Luque M, Sanz-Cabanillas JL, Alcalde-Mellado P, et al. Most systematic reviews of high methodological quality on psoriasis interventions are classified as high risk of bias using ROBIS tool. J Clin Epidemiol. 2017;92:79–88. 28893571 10.1016/j.jclinepi.2017.08.015

[95] Hooper EJ, Pandis N, Cobourne MT, Seehra J. Methodological quality and risk of bias in orthodontic systematic reviews using AMSTAR and ROBIS. Eur J Orthod. 2021;43(5):544–50. 33723612 10.1093/ejo/cjaa074

[96] Habtewold TD, Alemu SM, Mohammed SH, Endalamaw A, Mohammed MA, Teferra AA, et al. Biomedical and public health reviews and meta-analyses in Ethiopia had poor methodological quality: overview of evidence from 1970 to 2018. J Clin Epidemiol. 2019;109:90–8. 30721723 10.1016/j.jclinepi.2019.01.011

[97] Jaca A, Ndze VN, Wiysonge CS. Assessing the methodological quality of systematic reviews of interventions aimed at improving vaccination coverage using AMSTAR and ROBIS checklists. Hum Vaccin Immunother. 2019;15(12):2824–35. 31348722 10.1080/21645515.2019.1631567 PMC6930111

[98] Marzo Castillejo M, Montano BA. The GRADE system in taking clinical decisions and the elaboration of recommendations and clinical practice guidelines. Aten Primaria. 2007;39(9):457–60. 17919395 10.1157/13109491 PMC7659499

