# Supplementary material for: The methodological quality assessment of systematic reviews/meta-analyses of chronic prostatitis/chronic pelvic pain syndrome using AMSTAR2
Source: BMC Med Res Methodol. 2023 Nov 27;23:281. doi: 10.1186/s12874-023-02095-0 (PMC10680214; doi:10.1186/s12874-023-02095-0)
Supplement: Supplementary file 1 — Additional file 1. [file 12874_2023_2095_MOESM1_ESM.docx]

**Additional file 1.** Search Strategy

PubMed:

(("Prostatitis"[Mesh]) OR (((((((((Prostatitis[Title/Abstract]) OR (Prostatitides[Title/Abstract])) OR (Chronic Prostatitis with Chronic Pelvic Pain Syndrome[Title/Abstract])) OR (Chronic Prostatitis/Chronic Pelvic Pain Syndrome[Title/Abstract])) OR (Chronic Pelvic Pain Syndrome[Title/Abstract])) OR (Chronic Prostatitis[Title/Abstract])) OR (Chronic Prostatitides[Title/Abstract])) OR (CP/CPPS[Title/Abstract])) OR (CPPS[Title/Abstract]))) AND (((("Meta-Analysis" [Publication Type] OR "Meta-Analysis as Topic"[Mesh] OR "Network Meta-Analysis"[Mesh]) OR "Systematic Review" [Publication Type]) OR "Systematic Reviews as Topic"[Mesh]) OR (((((((((((Meta-Analysis[Title/Abstract]) OR (Meta Analysis[Title/Abstract])) OR (Meta-Analyses[Title/Abstract])) OR (Meta Analyses[Title/Abstract])) OR (Network Meta-Analysis[Title/Abstract])) OR (Systematic Review[Title/Abstract])) OR (System Review[Title/Abstract])) OR (Evidence based review[Title/Abstract])) OR (Evidence-based review[Title/Abstract])) OR (System evaluation[Title/Abstract])) OR (Systematic evaluation[Title/Abstract])))

EMBASE:

#7 #5 AND #6

#6 #3 OR #4

#5 #1 OR #2

#4 'meta analysis':ab,ti OR 'meta analyses':ab,ti OR 'network meta-analysis':ab,ti OR 'systematic review':ab,ti OR 'system review':ab,ti OR 'evidence based review':ab,ti OR 'evidence-based review':ab,ti OR 'system evaluation':ab,ti OR 'systematic evaluation':ab,ti

#3 'meta analysis'/exp OR 'meta analysis (topic)'/exp OR 'network meta-analysis'/exp OR 'systematic review'/exp OR 'systematic review (topic)'/exp

#2 'chronic prostatitis':ab,ti OR 'chronic pelvic pain syndrome':ab,ti OR prostatitis:ab,ti OR prostatitides:ab,ti OR 'chronic prostatitis with chronic pelvic pain syndrome':ab,ti OR 'chronic prostatitides':ab,ti OR 'chronic prostatitis/chronic pelvic pain syndrome':ab,ti OR 'cp/cpps':ab,ti OR cpps:ab,ti

#1 'chronic prostatitis'/exp OR 'chronic pelvic pain syndrome'/exp

Cochrane Library:

#1 MeSH descriptor: [Prostatitis] explode all trees

#2 ("prostatitis"):ti,ab,kw (Word variations have been searched)

#3 ("prostatitides"):ti,ab,kw (Word variations have been searched)

#4 (chronic prostatitis with chronic pelvic pain syndrome):ti,ab,kw (Word variations have been searched)

#5 ("chronic prostatitis/chronic pelvic pain syndrome"):ti,ab,kw (Word variations have been searched)

#6 (chronic prostatitis):ti,ab,kw (Word variations have been searched)

#7 ("chronic pelvic pain syndrome"):ti,ab,kw (Word variations have been searched)

#8 (chronic prostatitides):ti,ab,kw (Word variations have been searched)

#9 ("CP-CPPS"):ti,ab,kw (Word variations have been searched)

#10 (CPPS):ti,ab,kw (Word variations have been searched)

#11 #1 OR #2 OR #3 OR #4 OR #5 OR #6 OR #7 OR #8 OR #9 OR #10
