# Supplementary material for: The methodological quality assessment of systematic reviews/meta-analyses of chronic prostatitis/chronic pelvic pain syndrome using AMSTAR2
Source: BMC Med Res Methodol. 2023 Nov 27;23:281. doi: 10.1186/s12874-023-02095-0 (PMC10680214; doi:10.1186/s12874-023-02095-0)
Supplement: Supplementary file 3 — Additional file 3. [file 12874_2023_2095_MOESM3_ESM.docx]

**Additional file 3.** Quality classification of the AMSTAR2

| Quality classification | Definition |
| --- | --- |
| high | None or only 1 non-critical item does not meet the requirement: the SRs/MAs provide an accurate and comprehensive summary of the results of the available studies that address the question of interest. |
| moderate | More than 1 non-critical item does not meet the requirement: the SRs/MAs have more than one weakness but no critical flaws. It may provide an accurate summary of the results of the available studies that were included in the review. |
| low | 1 critical item does not meet the requirement with or without non-critical item does not meet the requirement: the SRs/MAs have a critical flaw and may not provide an accurate and comprehensive summary of the available studies that address the question of interest. |
| critically low | More than 1 critical item does not meet the requirement, with or without non-critical item does not meet the requirement: the SRs/MAs have more than one critical flaw and should not be relied on to provide an accurate and comprehensive summary of the available studies. |

*Multiple non-critical weaknesses may diminish confidence in the review and it may be appropriate to move the overall appraisal down from moderate to low confidence.
