# Supplementary material for: The methodological quality assessment of systematic reviews/meta-analyses of chronic prostatitis/chronic pelvic pain syndrome using AMSTAR2
Source: BMC Med Res Methodol. 2023 Nov 27;23:281. doi: 10.1186/s12874-023-02095-0 (PMC10680214; doi:10.1186/s12874-023-02095-0)
Supplement: Supplementary file 4 — Additional file 4. [file 12874_2023_2095_MOESM4_ESM.docx]

**Additional file 4.** Reference list of excluded studies reviewed in full-text with reasons

**Not all CP/CPPS[1-54]**

1. Taha DE, Aboumarzouk OM, Koraiem IO, Shokeir AA. Antibiotic therapy in patients with high prostate-specific antigen: Is it worth considering? A systematic review. Arab journal of urology. 2020;18(1):1-8.

2. Fan Y, Guo D, Wei Q, Tang Z, Cao DH, Yang L, et al. Antibiotics has incapability of reducing unnecessary prostate biopsies: A meta-analysis involving 2,035 patients. International Journal of Clinical and Experimental Medicine. 2016;9(2):4958-73.

3. Parsons BA, Goonewardene S, Dabestani S, Pacheco-Figueiredo L, Yuan Y, Zumstein V, et al. The Benefits and Harms of Botulinum Toxin-A in the Treatment of Chronic Pelvic Pain Syndromes: A Systematic Review by the European Association of Urology Chronic Pelvic Pain Panel. European urology focus. 2021.

4. Panunzio A, Tafuri A, Mazzucato G, Cerrato C, Orlando R, Pagliarulo V, et al. Botulinum Toxin-A Injection in Chronic Pelvic Pain Syndrome Treatment: A Systematic Review and Pooled Meta-Analysis. Toxins. 2022;14(1).

5. Carinci AJ, Pathak R, Young M, Christo PJ. Complementary and alternative treatments for chronic pelvic pain. Current Pain and Headache Reports. 2013;17(2).

6. Wagner B, Steiner M, Huber DFX, Crevenna R. The effect of biofeedback interventions on pain, overall symptoms, quality of life and physiological parameters in patients with pelvic pain : A systematic review. Wiener klinische Wochenschrift. 2022;134(Suppl 1):11-48.

7. Doble A. An evidence-based approach to the treatment of prostatitis: is it possible? Current urology reports. 2000;1(2):142-7.

8. Fojecki GL, Tiessen S, Osther PJ. Extracorporeal shock wave therapy (ESWT) in urology: a systematic review of outcome in Peyronie's disease, erectile dysfunction and chronic pelvic pain. World journal of urology. 2017;35(1):1-9.

9. Capogrosso P, Frey A, Jensen CFS, Rastrelli G, Russo GI, Torremade J, et al. Low-Intensity Shock Wave Therapy in Sexual Medicine—Clinical Recommendations from the European Society of Sexual Medicine (ESSM). Journal of Sexual Medicine. 2019;16(10):1490-505.

10. Gewandter JS, Chaudari J, Iwan KB, Kitt R, As-Sanie S, Bachmann G, et al. Research Design Characteristics of Published Pharmacologic Randomized Clinical Trials for Irritable Bowel Syndrome and Chronic Pelvic Pain Conditions: An ACTTION Systematic Review. The journal of pain. 2018;19(7):717-26.

11. Toye F, Seers K, Barker K. A meta-ethnography of patients' experiences of chronic pelvic pain: struggling to construct chronic pelvic pain as 'real'. Journal of advanced nursing. 2014;70(12):2713-27.

12. Mjaess G, Karam A, Roumeguère T, Diamand R, Aoun F, McVary K, et al. Urinary microbiota and prostatic diseases: the key for the lock? A systematic review. Prostate cancer and prostatic diseases. 2022.

13. Schoeb DS, Schlager D, Boeker M, Wetterauer U, Schoenthaler M, Herrmann TRW, et al. Surgical therapy of prostatitis: a systematic review. World journal of urology. 2017;35(11):1659-68.

14. Hao D, Yurter A, Chu R, Salisu-Orhurhu M, Onyeaka H, Hagedorn J, et al. Neuromodulation for Management of Chronic Pelvic Pain: A Comprehensive Review. Pain and Therapy. 2022;11(4):1137-77.

15. Terzoni S, Ferrara P, Parozzi M, Colombani F, Mora C, Cilluffo S, et al. Nurses' role in the management of persons with chronic urogenital pelvic pain syndromes: A scoping review. Neurourology and urodynamics. 2022.

16. Roumeguère T, Sfeir J, Rassy EE, Albisinni S, Antwerpen PV, Boudjeltia KZ, et al. Oxidative stress and prostatic diseases (Review). Translational Vision Science and Technology. 2017;7(5):723-8.

17. Sokolakis I, Pyrgidis N, Neisius A, Gierth M, Knoll T, Rassweiler J, et al. The Effect of Low-intensity Shockwave Therapy on Non-neurogenic Lower Urinary Tract Symptoms: A Systematic Review and Meta-analysis of Preclinical and Clinical Studies. European urology focus. 2022;8(3):840-50.

18. Taoka R, Kakehi Y. The influence of asymptomatic inflammatory prostatitis on the onset and progression of lower urinary tract symptoms in men with histologic benign prostatic hyperplasia. Asian Journal of Urology. 2017;4(3):158-63.

19. Mishra VC, Browne J, Emberton M. Role of repeated prostatic massage in chronic prostatitis: a systematic review of the literature. Urology. 2008;72(4):731-5.

20. Iacovelli V, Bianchi D, Pletto S, Pacini P, Fede Spicchiale C, Finazzi Agrò E. The role of glycosaminoglycans in the management of chronic pelvic pain: a systematic review. Minerva urologica e nefrologica = The Italian journal of urology and nephrology. 2020;72(3):321-31.

21. Gallo L. Effectiveness of diet, sexual habits and lifestyle modifications on treatment of chronic pelvic pain syndrome. Prostate cancer and prostatic diseases. 2014;17(3):238-45.

22. de Pedro Negri AM, Ruiz Prieto MJ, Díaz-Mohedo E, Martín-Valero R. Efficacy of Magnetic Therapy in Pain Reduction in Patients with Chronic Pelvic Pain: A Systematic Review. International journal of environmental research and public health. 2022;19(10).

23. Bratchikov OI, Tyuzikov IA, Dubonos PA. Nutritional supplementation of the pharmacotherapy of prostate diseases. Research Results in Pharmacology. 2021;7(3):1-14.

24. Song G, Wang M, Chen B, Long G, Li H, Li R, et al. Lower Urinary Tract Symptoms and Sexual Dysfunction in Male: A Systematic Review and Meta-Analysis. Frontiers in Medicine. 2021;8.

25. Liu C, Jiang TT, Zhang C, Gao HL, Wang SY, Zhang MP, et al. [Efficacy, safety and cost of Qianlieshutong Capsules in the treatment of chronic prostatitis]. Zhonghua nan ke xue = National journal of andrology. 2019;25(5):444-50.

26. Wu P, Yan R, Chen D, L BH, Zhang YX, Lu XY, et al. [Efficacy and adverse effects of Ningmitai Capsules combined with antibiotics in the treatment of chronic prostatitis: A meta-analysis]. Zhonghua nan ke xue = National journal of andrology. 2021;27(3):249-55.

27. Wang CY, Han RF. [Acupuncture for chronic prostatitis: a meta-analysis]. Zhonghua nan ke xue = National journal of andrology. 2008;14(9):853-6.

28. Yang MG, Zhao XK, Wang XJ. Management of Chronic Prostatitis(CP) in China: A meta-analysis of Randomized Controlled Trials (RCTs). Chinese Journal of Evidence-Based Medicine. 2007;7(10):737-42.

29. Qiu MX, Xiong GB, Gong BS, Wang D, Wang JY, Zhang SW. Traditional Chinese medicine for prostatitis: A systematic review of randomized controlled trials. Chinese Journal of Evidence-Based Medicine. 2010;10(1):56-72.

30. Qiu MX, Xiong GB, Zhou SY, Wang D, Shao JC, Wang JY. [Qingrelishi-category Chinese medicine for chronic prostatitis: a systematic review]. Zhonghua nan ke xue = National journal of andrology. 2007;13(4):370-7.

31. Deng W, Du X, Zhou W, Mei X, Tian Y, Chen L, et al. Systematic review and meta-analysis: α-adrenergic receptor blockers in chronic prostatitis. Annals of palliative medicine. 2021;10(9):9870-8.

32. Hui-Juan C, Shi-Bing L, Jian-Ping L, Bin W, Hai-Song L, Ji-Sheng W, et al. Qian lie an suppository (prostant) for chronic prostatitis: A systematic review and meta-analysis of randomized controlled trials. Medicine. 2019;98(14):e15072.

33. Li C, Xu L, Lin X, Li Q, Ye P, Wu L, et al. Effectiveness and safety of acupuncture combined with traditional Chinese medicine in the treatment of chronic prostatitis: A systematic review and meta-analysis. Medicine. 2021;100(49):e28163.

34. Han P, Wei Q, Shi M, Wu JC, Peng GH, Yang YR. Prostant™ in the treatment of chronic prostatitis: A meta-analysis. Asian journal of andrology. 2004;6(4):385.

35. Wang Z, Yuan L, Wang Y, Yang B, Dong X, Gao Z. Efficacy and safety of Chinese herbal medicine for chronic prostatitis associated with damp-heat and blood-stasis syndromes: a meta-analysis and literature review. Patient preference and adherence. 2016;10:1889-902.

36. Jiang Y, Cui D, Du Y, Lu J, Yang L, Li J, et al. Association of anti-sperm antibodies with chronic prostatitis: A systematic review and meta-analysis. Journal of reproductive immunology. 2016;118:85-91.

37. Condorelli RA, Russo GI, Calogero AE, Morgia G, La Vignera S. Chronic prostatitis and its detrimental impact on sperm parameters: a systematic review and meta-analysis. Journal of endocrinological investigation. 2017;40(11):1209-18.

38. Langston ME, Horn M, Khan S, Pakpahan R, Doering M, Dennis LK, et al. A Systematic Review and Meta-analysis of Associations between Clinical Prostatitis and Prostate Cancer: New Estimates Accounting for Detection Bias. Cancer epidemiology, biomarkers & prevention : a publication of the American Association for Cancer Research, cosponsored by the American Society of Preventive Oncology. 2019;28(10):1594-603.

39. Jiang J, Li J, Yunxia Z, Zhu H, Liu J, Pumill C. The role of prostatitis in prostate cancer: meta-analysis. PloS one. 2013;8(12):e85179.

40. Xia MK, Hu ZZ, Yang SN, Zhang J. Meta-analysis on Ningmitai Capsule combined with antibiotics therapy for chronic prostatitis. Chinese Traditional and Herbal Drugs. 2015;46(11):1704-9.

41. Cui D, Han G, Shang Y, Mu L, Long Q, Du Y. The effect of chronic prostatitis on zinc concentration of prostatic fluid and seminal plasma: a systematic review and meta-analysis. Current medical research and opinion. 2015;31(9):1763-9.

42. Vasavada SR, Dobbs RW, Kajdacsy-Balla AA, Abern MR, Moreira DM. Inflammation on Prostate Needle Biopsy is Associated with Lower Prostate Cancer Risk: A Meta-Analysis. The Journal of urology. 2018;199(5):1174-81.

43. Jin C, Chen Z, Zhang J. Meta-analysis of the efficacy of Ningmitai capsule on the treatment of chronic prostatitis in China. Medicine. 2018;97(33):e11840.

44. Perletti G, Monti E, Magri V, Cai T, Cleves A, Trinchieri A, et al. The association between prostatitis and prostate cancer. Systematic review and meta-analysis. Archivio italiano di urologia, andrologia : organo ufficiale [di] Societa italiana di ecografia urologica e nefrologica. 2017;89(4):259-65.

45. Guan Q, Zheng Y, Wei X, Wang S, Su B, Yu S. The Effect of Flavonoids on Chronic Prostatitis: A Meta-analysis of Published Randomized Controlled Trials. Journal of the National Medical Association. 2019;111(5):555-62.

46. Moryousef J, Blankstein U, Curtis Nickel J, Krakowsky Y, Gilron I, Jarvi K. Overview of seminal fluid biomarkers for the evaluation of chronic prostatitis: a scoping review. Prostate cancer and prostatic diseases. 2021.

47. Dun RL, Tsai J, Hu XH, Mao JM, Zhu WJ, Qi GC, et al. A systematic review of cross-cultural adaptation of the National Institutes of Health Chronic Prostatitis Symptom Index. Health and quality of life outcomes. 2021;19(1):159.

48. Li AS, Van Niekerk L, Wong ALY, Matthewson M, Garry M. Psychological management of patients with chronic prostatitis/chronic pelvic pain syndrome (CP/CPPS): a systematic review. Scandinavian journal of pain. 2022.

49. Khattak AS, Raison N, Hawazie A, Khan A, Brunckhorst O, Ahmed K. Contemporary Management of Chronic Prostatitis. Cureus. 2021;13(12):e20243.

50. Grinberg K, Sela Y, Nissanholtz-Gannot R. New insights about chronic pelvic pain syndrome (CPPS). International journal of environmental research and public health. 2020;17(9).

51. Harris-Hayes M, Spitznagle T, Probst D, Foster SN, Prather H. A Narrative Review of Musculoskeletal Impairments Associated With Nonspecific Chronic Pelvic Pain. PM and R. 2019;11(S1):S73-S82.

52. Naber KG, Niggemann H, Stein G, Stein G. Review of the literature and individual patients' data meta-analysis on efficacy and tolerance of nitroxoline in the treatment of uncomplicated urinary tract infections. BMC Infectious Diseases. 2014;14(1).

53. Zhao S, Wang Y, Wu W, Yang S, Feng L, Tao F, et al. Nonalcoholic fatty liver disease and risk of prostatic diseases: Roles of insulin resistance. Andrologia. 2021;53(6):e14060.

54. Buddingh KT, Maatje MGF, Putter H, Kropman RF, Pelger RCM. Do antibiotics decrease prostate-specific antigen levels and reduce the need for prostate biopsy in type IV prostatitis? A systematic literature review. Canadian Urological Association journal = Journal de l'Association des urologues du Canada. 2018;12(1):E25-e30.

**No classification of prostatitis[55-59]**

55. Dennis LK, Lynch CF, Torner JC. Epidemiologic association between prostatitis and prostate cancer. Urology. 2002;60(1):78-83.

56. Ramon R, Yahya H, Setiawan MR. Association between prostatitis and risk of prostate cancer: A systematic review and meta-analysis. International Journal of Urology. 2020;27(SUPPL 1):88.

57. Ding H, Fan S, Zhang L, Hao Z, Liang C. Does prostatitis increase the risk of prostate cancer? A meta-analysis. International Journal of Clinical and Experimental Medicine. 2017;10(3):4798-808.

58. Chen JX, Hu LS. Traditional chinese medicine for the treatment of chronic prostatitis in China: a systematic review and meta-analysis. Journal of alternative and complementary medicine (New York, NY). 2006;12(8):763-9.

59. Zhang L, Wang Y, Qin Z, Gao X, Xing Q, Li R, et al. Correlation between Prostatitis, Benign Prostatic Hyperplasia and Prostate Cancer: A systematic review and Meta-analysis. Journal of Cancer. 2020;11(1):177-89.

**Not systematic review[60-62]**

60. Nickel JC, Touma N. α-Blockers for the Treatment of Chronic Prostatitis/Chronic Pelvic Pain Syndrome: An Update on Current Clinical Evidence. Reviews in urology. 2012;14(3-4):56-64.

61. Anderson RU, NathansoN BH. Pain: drug therapies for CP/CPPS: help or hype?. Nature reviews Urology. 2011;8(5):236-237.

62. Luzzi GA. Chronic prostatitis and chronic pelvic pain in men: aetiology, diagnosis and management. Journal of the European Academy of Dermatology and Venereology. J Eur Acad Dermatol Venereo. 2002;16(3):253-256.
