# Supplementary material for: The methodological quality assessment of systematic reviews/meta-analyses of chronic prostatitis/chronic pelvic pain syndrome using AMSTAR2
Source: BMC Med Res Methodol. 2023 Nov 27;23:281. doi: 10.1186/s12874-023-02095-0 (PMC10680214; doi:10.1186/s12874-023-02095-0)
Supplement: Supplementary file 5 — Additional file 5. [file 12874_2023_2095_MOESM5_ESM.docx]

**Additional file 5.** The characteristic of “PICO” of the included studies

| Study | Participants | Intervention | Control | Outcome |
| --- | --- | --- | --- | --- |
| Collins et al. 1999 | CP/CPPS  (III) | medications used to treat benign prostatic hyperplasia; anti-inflammatory medications; antibiotics; thermotherapy; and miscellaneous medications | placebo; sham intervention; active pharmacologic or device therapy for chronic abacterial prostatitis | primary outcomes: the efficacy of treatment in improving urologic symptom scale scores or global reports of urinary tract symptoms. secondary outcomes: changes in the prostate examination, uroflowmetry, urodynamics, analysis of urine, expressed prostatic secretions and seminal fluid, and prostate ultrasonography |
| Collins et al. 2000 | CP/CPPS | medications used to treat benign prostatic hyperplasia; anti-inflammatory medications; antibiotics; thermotherapy | placebo; sham; active pharmacologic or device therapy for chronic abacterial prostatitis | primary outcomes: the sensitivity and specificity of the diagnostic test, the efficacy of treatment in improving urologic symptom scale scores or global report of urinary tract symptoms. secondary outcomes: changes in the prostate examination, uroflowmetry, urodynamics, analysis of urine, expressed prostatic secretions and seminal fluid, and prostate ultrasonography |
| Collins et al. 2002 | CP/CPPS  (IIIA/IIIB) | Allopurinol | placebo or other medical therapies | change in patient-reported discomfort, investigator-graded prostate pain, leukocyte counts, and biochemical indices |
| Yang et al. 2006 | CP/CPPS  (III) | α-blockers | placebo | NIH-CPSI; IPSS; pain; urinary symptoms; QoL |
| Lee et al. 2007 | CP/CPPS | α-blockers (tamsulosin，alfuzosin，terazosin) | placebo | NIH-CPSI scores |
| Mishra et al. 2007 | CP/CPPS  (III) | α -blockers | control groups received a placebo drug | NIH-CPSI scores |
| Yang et al. 2008 | CP/CPPS | α-blockers; antibiotics; phytotherapy; Other therapies | placebo | NIH-CPSI scores |
| Anothaisintawee et al. 2011 | CP/CPPS  (IIIA/IIIB) | α-blockers; antibiotics; steroidal and nonsteroidal anti-inflammatory drugs; finasteride; glycosaminoglycans; phytotherapy; gabapentinoids | placebo | NIH-CPSI scores; IPSS; PSSI; Other pain and voiding questionnaires |
| Aboumarzouk et al. 2012 | CP/CPPS  (IIIA/IIIB) | pregabalin | placebo; analgesia; any other method of symptom relief | primary outcomes: NIH-CPSI scores. Secondary outcomes: improvement of LUTS; the side effects from pregabalin |
| Cohen et al. 2012 | CP/CPPS  (III) | α-blockers; antibiotics; NSAIDs; finasteride; the glycosaminoglycan pentosan polysulfate; mepartricin; Secale cereale pollen extract; pregabalin; acupuncture; aerobic exercise; ESWT; PTNS | placebo; sham | NIH-CPSI total scores; NIH-CPSI pain domain subscore; NIH-CPSI voiding domain subscore; NIH-CPSI quality of life domain subscore |
| Thakkinstian et al. 2012 | CP/CPPS  (IIIA/IIIB) | α -blockers (terazosin, doxazosin, tamsulosin, alfuzosin, silodosin); antibiotics (ciprofloxacin, levofloxacin, tetracycline); any medical intervention in which the mechanism of action of the drug was at least in part related to its anti-inflammatory or immune modulatory activity (steroidal and nonsteroidal anti-inflammatory drugs, glycosaminoglycans, phytotherapy and tanezumab); the combination of antibiotics and α -blockers | placebo | NIH-CPSI scores; response rates ( responder definition was 25%, 33% or 50% decreases in NIH-CPSI; or 4-6 unit score decreases in total NIH-CPSI from baseline) |
| Moldwin et al. 2013 | CP/CPPS | Alternative therapies such as dietary and lifestyle modifications, acupuncture, phytotherapy, trigger point release and myofascial physical therapy, stress/cognitive-behavioral therapy, and other supportive measures | Standard medical therapy such as nonsteroidal anti-inflammatories, alpha-blockers, antimicrobial therapy, and 5a-reductase inhibitors | VAS pain scores, NIH-CPSI scores, NIH-CPSI pain domain scores, and NIH-CPSI QoL domain scores |
| Fu et al. 2014 | CP/CPPS | NA | NA | semen volume; sperm concentration; total sperm count; sperm progressive motility; sperm total motility; sperm vitality; and sperm normal morphology |
| Riegel et al. 2014 | CP/CPPS（IIIA/IIIB） | NA | NA | Psychosocial factors (pain catastrophizing cognitions, stress, personality factors, and social aspects/relationship functioning); Psychiatric co-morbidity (any psychiatric diagnosis according to DSM-IV/ ICD-10 or self-report questionnaires measuring psychopathology); QoL |
| Zhu et al. 2014 | CP/CPPS（III） | Antibiotics (levofloxacin, mepartricin, ciprofloxacin, tetracycline) | placebo and others (Tamsulosin, Doxazosin) | CPSI score, which includes pain score, voiding score, QoL score, and total score |
| Chen et al. 2015 | CP/CPPS | NA | NA | Number of ED patients; IIEF-5; NIH-CPSI |
| Li et al. 2015 | CP/CPPS | NA | NA | the prevalence of sexual dysfunction |
| Liu et al. 2016 | CP/CPPS | acupuncture; electroacupuncture; catgut embedding | sham acupuncture; standard medicine | total NIH-CPSI score; response rate; subscores of NIH-CPSI; IPSS; laboratory indicators; adverse events |
| Qin et al. 2016a | CP/CPPS  (III) | electro-acupuncture, acupuncture, α-blockers, antibiotics, combination of alpha-blockers or antibiotics or NSAIDs | sham acupuncture; placebo | NIH-CPSI scores |
| Qin et al. 2016b | CP/CPPS（III） | electro-acupuncture; manual acupuncture; manual acupuncture plus medicine | drugs; penetrating acupuncture on non-acupuncture points | total NIH-CPSI scores; NIH-CPSI subscale score; IPSS; the global response rate |
| Cai et al. 2017 | CP/CPPS | pollen extract | Placebo; ibuprofen; Eviprostat (phytotherapeutic agent) | NIH-CPSI score; SF-36 questionnaires; IPSS |
| Chang et al. 2017 | CP/CPPS（IIIA/IIIB） | acupuncture | Sham acupuncture; α-blockers;  antibiotics; non-steroidal anti-inflammatory agents | response rate; NIH-CPSI scores; IPSS |
| Anderson et al. 2018 | CP/CPPS | physical therapy; biofeedback; cognitive behavioral therapy | drug therapies | NIH-CPSI scores |
| Franco et al. 2018 | CP/CPPS（III） | nonpharmacological interventions (acupuncture; lifestyle modifications; physical activity; prostatic massage; ESWT; transrectal thermotherapy; other interventions) | placebo; sham procedure; pharmacological interventions | Prostatitis symptoms; Adverse events; SD; Urinary symptoms; QoL; depression and anxiety |
| Franco et al. 2019 | CP/CPPS  (III) | pharmacological interventions (α-blockers; 5-alpha reductase inhibitors; antibiotics; Anti-inflammatories; Phytotherapy; Botulinum toxin A; Allopurinol; Traditional Chinese medicine) | placebo; other types of pharmacological interventions | Prostatitis symptoms; Adverse events; Sexual dysfunction; QoL; Depression and anxiety; Urinary symptoms |
| Liao et al. 2019 | CP/CPPS | (ESWT) | placebo; other treatments | NIH-CPSI scores; the rate of overall clinical effectiveness |
| Qin et al. 2019a | CP/CPPS | acupuncture | sham acupuncture | NIH-CPSI scores |
| Qin et al. 2019b | CP/CPPS | acupuncture | sham acupuncture | primary outcome: the response rate of acupuncture. secondary outcomes: NIH-CPSI total score; NIH-CPSI subscale scores |
| Yuan et al. 2019 | CP/CPPS | LI‐ESWT | sham therapy | NIH‐CPSI scores; Qmax; PVR |
| Birowo et al. 2020 | CP/CPPS | ESWT | placebo; sham | VAS; urinary score; QoL; NIH-CPSI scores |
| Huang et al. 2020 | CP/CPPS | NA | NA | the severity or the prevalence of psychological factors and pain catastrophizing of patients |
| Li et al. 2020 | CP/CPPS（III） | Acupuncture | sham acupuncture; medication (such asα-blockers, antibiotics, or anti-inflammatory drugs) | total NIH-CPSI score; NIH-CPSI subscale score; IPSS; global response rate; adverse events |
| Chen et al. 2021 | CP/CPPS | NA | NA | levels of IL1β, IL-2, IL-4, IL-6, IL-8, IL-10, TNF-α, IFN-γ, macrophage inflammation protein 1α (MIP-1α), and the white blood cell (WBC) count |
| Kang et al. 2021 | CP/CPPS | ESWT; acupuncture | sham procedure | total NIH-CPSI score; NIH-CPSI subscale score; IPSS; IIEF score; response rate; adverse events |
| Li et al. 2021 | CPPS | LI-ESWT | drug treatment; sham control | total NIH-CPSI scores, QoL, VAS scores, and urinary symptoms |
| Mykoniatis et al. 2021 | CP/CPPS（IIIB） | LI-ESWT | sham therapy | total NIH-CPSI scores; QoL scores; IPSS; IIEF; PVR; Qmax |
| Zhang et al. 2021a | CP/CPPS（IIIA/IIIB） | acupuncture | sham acupuncture; standard medication; acupuncture plus standard medication | primary outcome: the change of total NIH-CPSI scores. secondary outcomes: the changes in NIH-CPSI subscale score |
| Zhang et al. 2021b | CP/CPPS with SD | OCPM plus WY | WM alone or another OCPM plus WM | NIH-CPSI; IIEF-5; the clinical effective rate of CP/CPPS; the clinical effective rate of SD |
| Kong et al. 2022 | CP/CPPS | LI-ESWT; LI-ESWT combined with medications | Sham; sham combined with medications; medications alone | NIH‐CPSI scores; VAS; IIEF; IPSS |
| Lao et al. 2022 | CP/CPPS（IIIA/IIIB） | selective serotonin reuptake inhibitors (SSRIs)alone; SSRI combined with other routine interventions | Placebo; another SSRI; routine interventions | NIH-CPSI scores; SDS scores; the clinical effective rate of CP/CPPS; IELT |
| Lok et al. 2022 | CP/CPPS | Serenoa repens | Placebo; Other drugs | NIH-CPSI scores |
| Andrey et al. 2022 | CP/CPPS | Placebo | Symptoms before and after treatment | NIH‐CPSI score, IPSS score, Qmax, PVR |
| Qin et al. 2022a | CP/CPPS | acupuncture; acupuncture plus medication | sham acupuncture; medication | NIH-CPSI total score; NIH-CPSI pain domain score; NIH-CPSI urinary domain score; NIH-CPSI QoL domain score; global response rate; IPSS; adverse events |
| Qin et al. 2022b | CP/CPPS | oral pharmacological treatments | oral pharmacological treatments; Placebo | total NIH-CPSI scores; NIH-CPSI pain score; NIH-CPSI urinary score; NIH-CPSI QoL score; adverse events |
| Zhao et al. 2022 | CP/CPPS（III） | NA | NA | abnormal brain areas and trends in CP/ CPPS, coordinates, and clinical correlation |

NIH-CPSI, National Institutes of Health Chronic Prostatitis Symptom Index; IPSS, International Prostate Symptom Score; PSSI, Prostatitis Symptom Score Index; QoL, Quality of life; IIEF-5, International Index of Erectile Function-5; Qmax, maximum urinary flow rate; PVR, post-void residual volume; VAS, visual analog scale; SD, sexual dysfunction; ED, erectile dysfunction; SDS, Self-Rating Depression Scale; IELT, intravaginal ejaculation latency time; NSAIDs, non-steroidal anti-inflammatory medications; ESWT, extracorporeal shock wave therapy; PTNS, percutaneous posterior tibial nerve stimulation; LI‐ESWT, low‐intensity extracorporeal shock wave; OCPM, Oral Chinese Patent Medicine; WY, western medicine; NA, not applicable.
