# Supplementary material for: The methodological quality assessment of systematic reviews/meta-analyses of chronic prostatitis/chronic pelvic pain syndrome using AMSTAR2
Source: BMC Med Res Methodol. 2023 Nov 27;23:281. doi: 10.1186/s12874-023-02095-0 (PMC10680214; doi:10.1186/s12874-023-02095-0)
Supplement: Supplementary file 6 — Additional file 6. [file 12874_2023_2095_MOESM6_ESM.docx]

**Additional file 6.** Sensitivity analysis based on different definitions of publication year of all included studies (N = 45)

| Characteristics | Coefficients | 95% CI | P value |
| --- | --- | --- | --- |
| Publication year (Before 2018) |  |  |  |
| After 2018 | 0.161 | -0.235, 0.556 | 0.415 |
| Continent (Asia) |  |  |  |
| Europe | 0.515 | -0.012, 1.041 | 0.055 |
| North America | -0.092 | -0.596, 0.411 | 0.712 |
| South America | -0.406 | -1.516, 0.704 | 0.462 |
| PRISMA (No) |  |  |  |
| Yes | -0.055 | -0.457, 0.347 | 0.782 |
| Preregistration (No) |  |  |  |
| Yes | 0.217 | -0.159, 0.594 | 0.249 |
| Funding support (No) |  |  |  |
| Yes | -0.109 | -0.529, 0.309 | 0.598 |
| RCT enrollment (non-RCTs) |  |  |  |
| RCTs and non-RCTs | -0.290 | -0.837, 0.257 | 0.289 |
| RCTs | -0.207 | -0.664, 0.251 | 0.364 |
| Whether CDSR (non-CDSR) |  |  |  |
| CDSR | 0.571 | -0.185, 1.327 | 0.134 |
| Meta-analysis (Without) |  |  |  |
| With | 0.107 | -0.449, 0.663 | 0.698 |
