# Supplementary material for: The methodological quality assessment of systematic reviews/meta-analyses of chronic prostatitis/chronic pelvic pain syndrome using AMSTAR2
Source: BMC Med Res Methodol. 2023 Nov 27;23:281. doi: 10.1186/s12874-023-02095-0 (PMC10680214; doi:10.1186/s12874-023-02095-0)
Supplement: Supplementary file 7 — Additional file 7. [file 12874_2023_2095_MOESM7_ESM.docx]

**Additional file 7.** Sensitivity analysis based on different definitions of publication year of interventional SRs/MAs of CP/CPPS (N = 38)

| Characteristics | Coefficients | 95% CI | P value |
| --- | --- | --- | --- |
| Publication year year (Before 2018) |  |  |  |
| After 2018 | 0.111 | -0.376, 0.598 | 0.642 |
| Continent (Asia) |  |  |  |
| Europe | 0.715 | 0.088, 1.343 | 0.027 |
| North America | -0.008 | -0.613, 0.596 | 0.978 |
| South America | -0.122 | -1.406, 1.163 | 0.847 |
| PRISMA (No) |  |  |  |
| Yes | -0.051 | -0.528, 0.425 | 0.827 |
| Preregistration (No) |  |  |  |
| Yes | 0.149 | -0.311, 0.610 | 0.511 |
| Funding support (No) |  |  |  |
| Yes | 0.033 | -0.460, 0.526 | 0.892 |
| RCT enrollment (non-RCTs) |  |  |  |
| RCTs and non-RCTs | -0.290 | -1.392, 0.811 | 0.592 |
| RCTs | -0.176 | -1.320, 0.968 | 0.755 |
| Whether CDSR (non-CDSR) |  |  |  |
| CDSR | 0.457 | -0.460, 1.374 | 0.315 |
| Meta-analysis (Without) |  |  |  |
| With | 0.123 | -0.686, 0.932 | 0.758 |
